# DeepConf: Leveraging ANI-ML Potentials for Exploring Local Minima with Application to Bioactive Conformations

**DOI:** 10.1021/acs.jcim.4c02053

**Published:** 2025-03-04

**Authors:** Omer Tayfuroglu, Irem N. Zengin, M. Serdar Koca, Abdulkadir Kocak

**Affiliations:** Department of Chemistry, Gebze Technical University, 41400 Kocaeli, Turkey

## Abstract

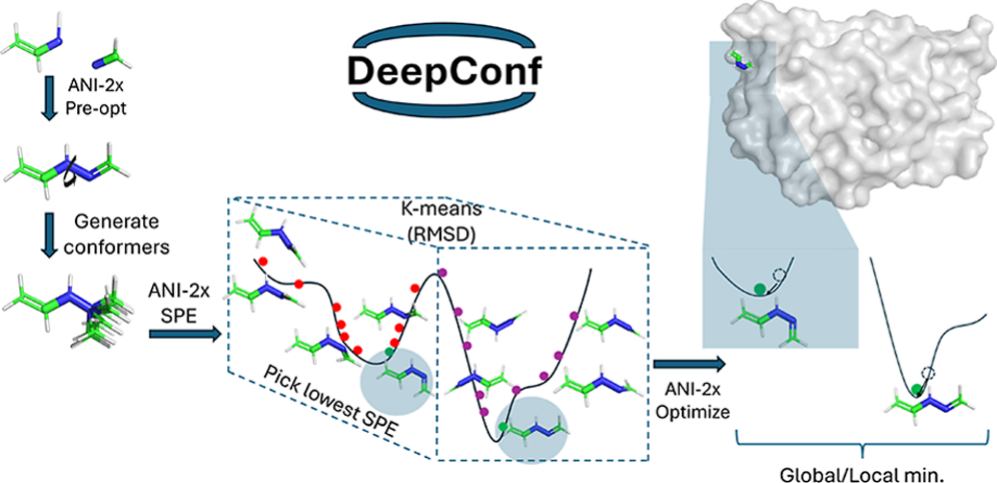

Here, we introduce a low energy conformer generation algorithm using ANI-ML potentials at the DFT accuracy and benchmark in reproducing bioactive conformations. We show that the method is efficient when the initial starting structure is far from equilibrium, when the ML potentials are stuck in nonsmooth regions, and when the quality of the conformers in a less conformer size is demanded. We specifically focus on conformations due to rotations around the single bonds. For the first time, we assess the performance of ANI-ML potentials using our conformer generation algorithm, DeepConf, in addition to previously reported Auto3D (*J. Chem. Inf. Model.***2022**, *62*, 5373–5382) using the same potentials to reproduce bioactive conformations as well as providing a guideline for bioactive conformation evaluation processes. Our results show that the ANI-ML potentials can reproduce the bioactive conformations with mean value of the root-mean-square-deviation (RMSD) less than 0.5 Å, outperforming the limit of conventional methods. The code offers several features including but not limited to geometry optimization, fast conformer generations via single point energies (SPE), different minimization algorithms, different ML-potentials, or high-quality conformers in the smallest amount of ensemble sizes. It is available free of charge (documentation and test files) at https://github.com/otayfuroglu/DeepConf.

## Introduction

Isomerism (as constitutional isomerism and stereoisomerism) play a key role in physical organic chemistry as well as drug design and discovery.^1,2^ Experimental and computational methods can easily elucidate the structural (constitutional) isomers since their physical and chemical properties are different due to different connectivity.^3^ Similarly, configurational stereoisomers (around a double bond) usually require remarkably high energy for interconversion, making them easy to model computationally. For organic chemists, conformational isomers may not be a major concern since 2D representation is mostly sufficient to depict synthesized compounds. Likewise, experimental procedures for synthesis and characterization are mostly performed at the macroscopic level, so all the accessible conformational isomers are already reflected on the measurements as an equilibrated mixture under experimental conditions (e.g., room or physiological temperature, solution phase, etc.). However, for computer-aided drug design studies (CADD), conformational stereoisomers (conformers) around single rotatable bonds (i.e., torsion points) can become cumbersome due to their vast number of energetically accessible rearrangements, making the true 3D representation of a molecule vital. Indeed, most drug-like organic compounds contain multiple rotatable bonds, creating a conformational space that must be included in computational studies.^4,5^

The energy change in response to the dihedral angle change around the four adjacent atoms in a single (rotatable) bond can be too small to accurately predict by computational methods. Even if the dihedral angle is predicted accurately, it only represents one of the many possible conformers (local minima). Finding the true 3D structure with the lowest energy of all conformers (global minimum) requires the energies of all minima to be calculated accurately. As a further complication in computer aided drug design, all conformers need to be evaluated at their biological target, as the most stable complex between a biological target molecule such as proteins and a drug-like molecule (i.e., the bound state) may not necessarily correspond to the global minimum of the drug-like compound in the free (unbound) state.^4^ Moreover, the structural strain on the bioactive compound, which is relatively small at the torsion points, can be easily outweighed by the possible strong interactions with the biotarget compound, leading to the bound state of drug-like compounds possessing a nonequilibrium structure. For this reason, most computational tools, such as molecular docking, allow free rotation around these torsion points and use the ligand strain energy in the binding affinity calculations as a compromise to the energy gain between the bound and the unbound states.

Indeed, there is an entire field in computational chemistry dedicated to the accurate and fast prediction of binding free energy (BFE) changes upon ligand binding to its biological target compound.^6−41^ Thus, besides finding low energy conformers of a drug-like compound, it is also necessary to find the relative energies among the conformers and potential energy surface (PES) connecting these conformations. Numerous strategies, beyond the screening of conformers using molecular docking, have been developed, including induced-fit docking, ensemble docking, molecular dynamics (MD) and Monte Carlo (MC) simulations, all of which are involved at some level in the conformational space of the ligand in BFE calculations.

Most of the concern in isomerism arises from conformational isomers due to torsional points around single bonds, as almost all the conformational isomers are energetically accessible and populated to some extent. Thus, the number of torsion points defines (and mostly limits) the number of conformational isomers to be sampled to obtain more accurate energetics of the molecule. The best scenario for examining low energy lying conformations is to systematically calculate all possible conformations and then find all local minima along with the global minimum structure. However, as the number of torsion points increases, the number of different conformations to assess grows exponentially. For instance, a simple flip (180° rotation) around a single bond creates two possible conformations to evaluate, and if there is six such torsion points in a small molecule, a total of 2^6^ = 64 calculations (minima on PES) must be completed to identify the lowest energy (global minimum) and other low-energy (local minima) structures. For this reason, most conformer generation algorithms use stochastic search algorithms rather than systematically evaluating all conformers.

Ideally, all minima on the PES should be produced by multidimensional (3N-6) scanning of all coordinates at the most accurate quantum mechanical (QM) levels. Otherwise, a systematic evaluation of all possible conformers at the most accurate level is required to gain insight into the global minimum and all local minima, along with their relative energies and populations. Since there are energy barriers between different minima on the PES, minimization algorithms using energy gradients (i.e., minimizing energy with respect to coordinates) only find the nearest minimum where the energy gradient (force) reaches to the zero threshold. Thus, an extensive search with different initial conformations is still required to locate all minima.

Over the years, numerous commercial (e.g., iCon,^42^ Omega,^43^ ConfGen,^44^ MOE,^45^ CREST^46^) and open-source tools (e.g., FROG2,^47,48^ Confab,^49^ OpenBabel,^50^ and RDKit^51^) have been developed for fast and accurate conformer generation. Most recently, Seidel et al.^4^ and earlier Friedrich et al.^52^ reviewed the performance of some of these software tools. Due to the enormous number of conformer calculations, most algorithms use molecular mechanics (MM)-based minima searches rather than more accurate QM methods. However, searching for minima using MM methods introduces additional problems. First, they rely on the accuracy of the force field (FF) used and to cover all chemical library, they need to be generalized (such as UFF, GAFF, MMFF94), which results in a loss of accuracy. Second, they use empirical parameters for bonds, angles, and dihedral definitions for covalent interactions, as well as fixed partial atomic charges and Lennard-Jones parameters for noncovalent interactions. They require well-defined bonding information in the initial structure. If the initial structure is incorrectly drawn (by the user) or the preassigned bond order is incorrect, MM based minimization algorithms may totally fail to locate the correct nearest minimum and other minima. Furthermore, even if the bonding is correctly defined and the local minimum is in the correct conformation, the relative energies are still in MM level, which might deviate from the actual values. For these reasons, there is a need to perform minimizations and search for conformers at QM accuracy. Searching for global and local minima using QM methods such as DFT can become computationally prohibitive due to the enormous number of conformation evaluations.

One strategy to balance QM accuracy and computational cost is to use machine learning (ML) techniques to model the multidimensional PES of the system at QM accuracy. To date, several neural network potentials (NNPs) have been developed to represent the multidimensional PES of systems at QM accuracy. In principle, if the PES of the system is well-represented by NNPs, the energy minimization and conformer search can be performed using ML potentials rather than costly QM calculations or less accurate MM calculations.

The most popular ML potentials are ANI^53−56^ (with versions of ANI-1, ANI-2x, and ANI-2xt) and AIMNET, which have been successfully utilized for energy minimization and low-lying minimum search using several gradient based minimization algorithms.^3,57^ A general issue with ML potential is that the PES is not expressed in an explicit mathematical function form. As a result, its smoothness depends heavily on the quality of the training data, and it may not be as smooth or physically interpretable as the PES from QM or MM methods. Having a nonsmooth PES raises problems in gradient-based minimizations as they can easily be stuck on saddle-points where the energy derivation with respect to coordinate is zero. While such challenges are inherent to both smooth and nonsmooth potential energy surfaces (PES), nonsmoothness worsens the problem by introducing abrupt discontinuities in PES, often arising from undertrained ML models with insufficient or low-quality data. This issue is particularly pronounced for geometries far from equilibrium, where sampling is sparse.

In addition, drawing errors in typical chemical software (e.g., ChemDraw, MarvinSketch, GaussView, or Spartan) can lead to incorrect bond types, misassigned connectivity (e.g., single bonds instead of double bonds or missing ring closures), or improper bond lengths. While these programs generally provide “cleaning” utilities based on molecular mechanics (MM), errors persist if the connectivity is initially misdefined.

Herein, we attempt to improve the minimization and local minima search based on ANI-ML potentials by introducing a strategy that generates multiple conformations, clusters them according to RMSD, and refines the representative conformer in each cluster. This multiconformer approach broadens the search space and reduces the likelihood of the optimization becoming trapped in local minima, particularly in nonsmooth regions of the PES. We start with small systems as a case study to overcome nonsmooth regions, demonstrating that our conformer generation algorithm allows for successfully reaching local minima. Then, we extend our approach to a larger data set, where bioactive conformations are generated using ANI-ML potentials, and evaluate its performance on this broader scope.

### Computational Methods

In the QM calculations, we used Gaussian 16 software with the WB97X/6-31G* level since ANI-2x is trained at this DFT level. Custom Python scripts along with Openbabel and RDKit were used extensively in RMSD calculation, file conversion, and conformer generation processes. Corresponding GitHub repositories have been used in Auto3D calculations. The atomic simulation environment (ASE) was used in our custom script for the energy minimizations. The details and usage of our custom script for conformer generations can be accessed freely via https://github.com/otayfuroglu/DeepConf.

## Results and Discussion

### Workflow

**Figure 1:**
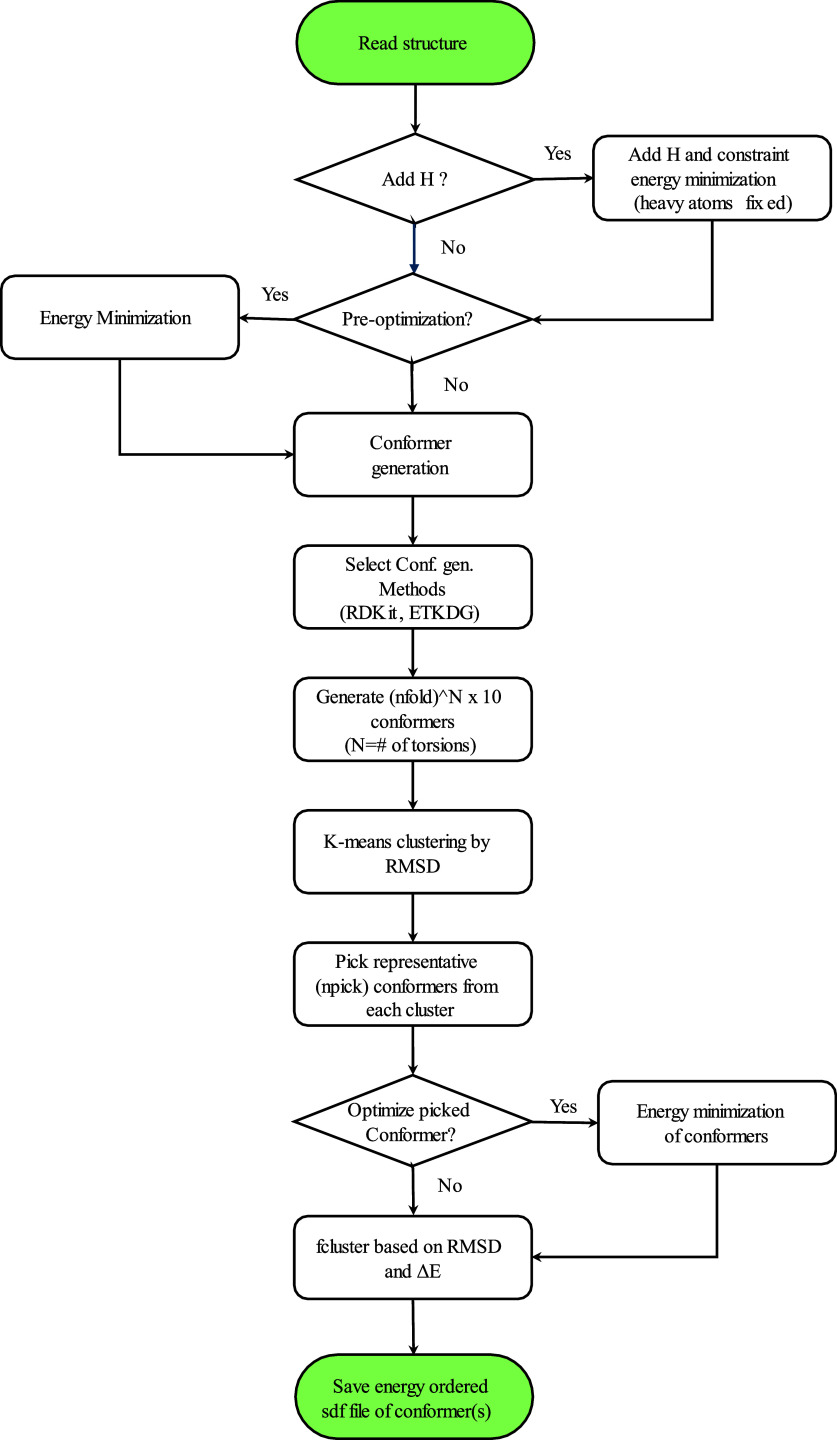
DeepConf flowchart.

DeepConf is written in Python 3.8 and uses ASE libraries to calculate energy and force components in the conformer generations and energy minimization processes. The workflow for the prediction of local minima and conformer generation is given in Figure 1.

The program starts reading the common 3D files (xyz, mol2, sdf and pdb file formats) from a directory of the ligands and fixes the structure by adding required hydrogen atoms if needed, upon user request. Depending on the user’s choice, preoptimization of the ligand is performed using several optimization methods (optimization_method = BFGS, LBFGS, FIRE, GPMin or Berny) and the energy/force calculator (calculator_type = ANI-2x or G16) until the convergence threshold is achieved (set by thr_fmax and maxiter, corresponding to maximum force and maximum iterations). Then, the program identifies rotatable bonds (torsion points) using the RDKit library, with or without the ETKDG approach. After identifying the torsion points, the program requests the RDKit/ETKDG to sample diverse conformations. To reduce redundancy and select representative structures, the generated conformers are clustered based on root-mean-square deviation (RMSD) of atomic coordinates using *K*-means clustering implemented in the SciPy library. The lowest-energy structures in each cluster are chosen as the final representative conformations. This approach ensures that the final set of conformers is both diverse and energetically favorable. To enhance conformer diversity, the program uses the nfold parameter to request a specified number of conformers from ETKDG, with the default set to 2. This generates a total of (nfold)^*N*^ × 10 conformations, where *N* represents the number of torsions. It is important to note that chemical preferences for torsions (e.g., C(sp3)–C(sp3) favoring ∼60°/120°) are handled by ETKDG, regardless of the nfold value. For instance, if there are 5 torsions and each one defined to flip 180° (i.e., nfold = 2), a total of 2^5^ × 10 = 320 structures are produced. Additionally, the maximum number of conformations can be set by the user to avoid generating too many conformations. Next, the program generates (nfold)^*N*^ different clusters, where *N* represents the number of torsions, using *K*-means clustering and distributes generated structures into these clusters to ensure that each fold is categorized into a distinct cluster. For example, for a molecule with *N* = 5 torsions and nfold = 2, the program generates 2^5^ = 32 clusters and distributes the 320 generated structures among them. Then, it calculates the single-point energies (SPE) of the structures and selects one representative structure (the one with the lowest energy) from each cluster. While the conformations are expected to be physically valid within the limits of RDKit/ETKDG, some unphysical structures, such as atom clashes, could arise. However, the SPE calculations, combined with clustering, effectively eliminate these unphysical structures, as they will have significantly higher energies compared to other conformers in the cluster. At this point, the user can set extra structures for consideration from each of the clusters (default is npick = 0, corresponding to no additional structure pick from clusters and pick only the lowest energy one). In addition, if the user wants to optimize the picked structures from cluster, the program uses the minimization methods and calculators to minimize the structures. The last part is to eliminate the structures that are converging to the same equilibrium geometries. This is done by using F-clustering, a hierarchical clustering method implemented in the SciPy library, based on RMSD (opt_prune_rms_thresh) and energy (opt_prune_diffE_thresh) thresholds. The final structures represent the global and local minima structures.

The program offers flexibility in searching for conformers along with geometry optimizations. One could just perform geometry optimizations by turning off the conformer generation process or rapidly generate conformers without geometry optimizations on picked conformers. Alternatively, one could search for all local minima along with the global minimum by turning on the optimization on the picked conformers.

We can highlight the solutions the program provides to a few complications encountered in conventional conformer generation algorithms:

1 if the initial starting structure is not drawn correctly (by the user) or bonding is incorrect (by the user or the MM predefinitions for bond orders), our script fixes that by performing preoptimization process, which is not affected by bonding information.
2 If the user wants to obtain the most plausible conformations among different torsions as quickly as possible, the program selects the best torsion angles corresponding to the lowest-energy structure purely based on single-point energies (SPE), even without further optimizations.
3 If the user would like to generate most local minima extensively with the smallest ensemble size, the program attempts to find them all smartly using the flowchart given in Figure 1.
4 If the optimization is stuck due to nonsmoothness of the ML potential, the program attempts to find several other points that might lead to better solutions for the energy minimization problem.

### ANI-ML Potentials for PES

**Figure 2:**
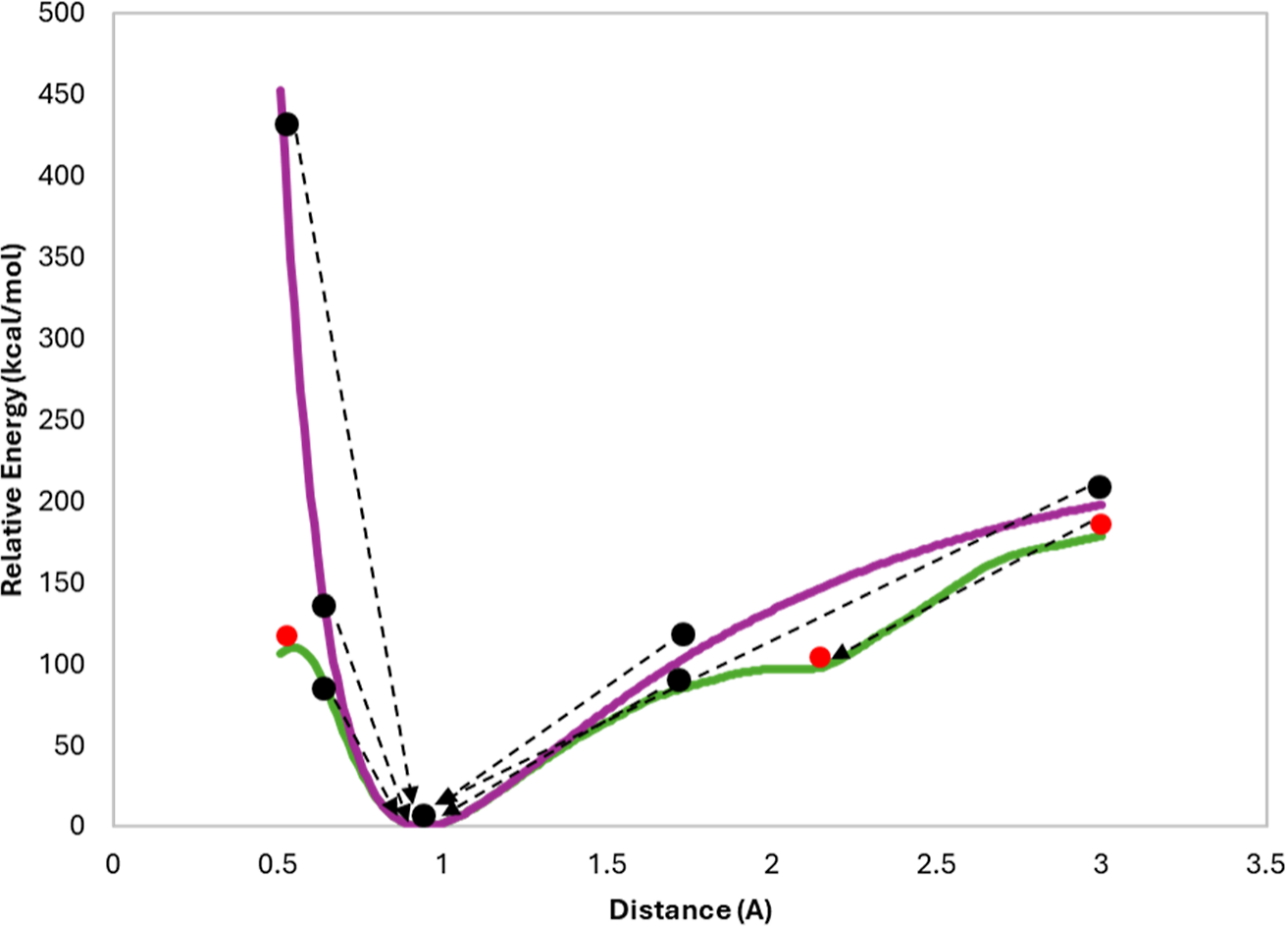
PES scan of the H–F distance computed using G16 (blue) and ANI-2x (green). Black and red spheres, respectively, represent the initial structures reaching the equilibrium geometry and failing to converge during gradient-based optimization.

ANI-ML potentials can stop earlier than the real minimum reached because of the nature of the nonsmooth PES. We start with hydrogen fluoride (HF), a diatomic molecule with one of the simplest representations of PES (only on H–F distance), as an illustration of how ML-potentials can be nonsmooth. Figure 2 shows the potential energy profile of H–F distance calculated by G16 and ANI-2x. If one were to perform a geometry optimization of HF starting with an initial structure of H–F distance outside the 0.6–1.2 Å range, the optimization with ANI-2x would fail due to saddle points near 0.5 and 1.9 A whereas G16 still brings the structure to the equilibrium distance of 0.96 Å. Indeed, we started at several different initial distances and observed convergence of energy at the saddle point in the case of LBFGS algorithm and ANI-2x calculator. Although it is not the case in this study, it should be noted that ANI is trained on neutral compounds and is not capable of accurately modeling spin states or charges that may arise when atomic distances exceed the bond dissociation limit.

**Figure 3:**
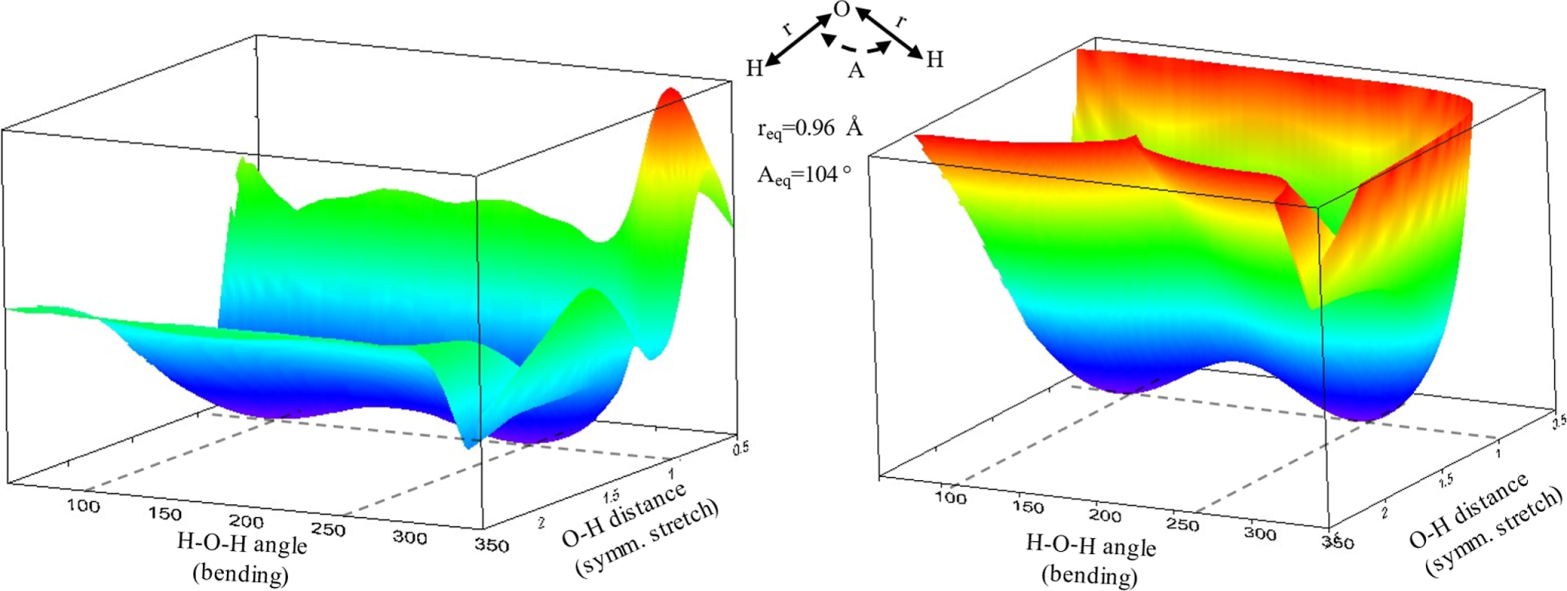
PES of H–O–H angle and O–H bond distances by ANI-2x (left) and G16 (right).

To better illustrate when the system is larger, we also performed a two-dimensional scan (the symmetric stretching and bending vibrational modes) of the coordinates of H_2_O. Figure 3 shows the nonsmooth regions on PES generated by ANI-2x.

In larger compounds with several torsion points, the multidimensional PES often contains numerous nonsmooth regions. Our approach begins with several diverse points on this representative PES, generated by RDKit/ETKDG to more efficiently converge to equilibrium geometries.

When structures are near equilibrium, ANI-ML potentials perform well in locating the nearest minimum. However, when far from equilibrium, ANI-ML potentials can become stuck in nonsmooth regions of the multidimensional PES. These nonsmooth regions occur more frequently in dihedral angles because ANI-ML potentials are not trained to include interactions beyond 1–4. Previous studies have reported significant deviations of ANI-ML potentials from QM calculations at torsional energy barriers.^58^

**Figure 4:**
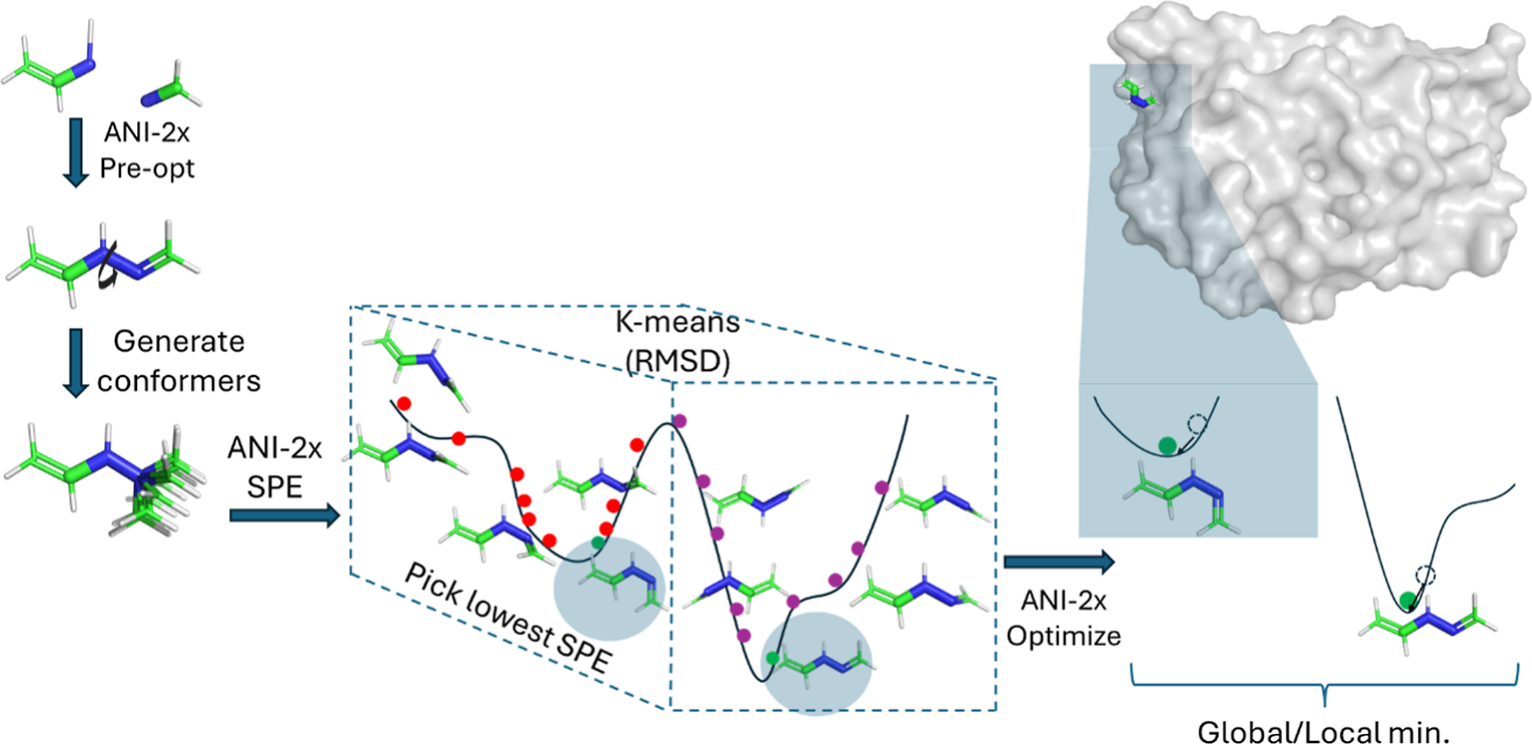
DeepConf’s approach to overcoming optimization challenges in nonsmooth regions of the potential energy surface.

Our approach addresses this issue by creating diverse solutions at torsional points. This is achieved by boosting RDKit/ETKDG conformations, followed by selecting the lowest-energy structure from this diverse set. This strategy ensures that even in challenging regions of the PES, a structure has more opportunities to reach the nearest local minimum within the smallest ensemble size possible (Figure 4).

Our first step with ANI is to assess it on several small compounds, in which there are only a few torsion points. Using the compounds in Figure S1, we first manually generated the most plausible conformers including intermediate dihedral angles (up to 200 conformations for each compound) via rotation on each torsion. Next, we optimized each structure at the WB97X/6-31G* level to create our reference structures of global minimum and other possible local minima that the QM calculations predict by eliminating structures that converge to the same equilibrium conformers. These reference structures were used to assess the performance of DeepConf and Auto3D, both utilize ANI-ML potentials.

When we used the manually generated conformations by rotating around the torsion points (i.e., including several intermediates at each torsion point), ANI based optimizations yield the same conformers predicted by QM calculations (Table 1). This is as expected because we manually generated intermediate structures connecting each minimum and thus no conformer generation algorithm is required (i.e., DeepConf and Auto3D are the same at this point as ANI-2x). However, the extensive sampling for each torsion via several different dihedral angles eventually leads to ANI covering all the G16 minima. This also indicates that even if ANI can be stuck in a nonlocal geometry due to nonsmooth PES, the optimization problem can be overcome by taking several samples from intermediate structures and optimizing them.

**Table 1:** ANI Predicted the Number of Minima and Its Success in Terms of Reproducing G16 Predicted Ones from Manually Created Intermediate Conformations for Each Torsion Point

ID	G16 number of minima	ANI number of minima	number of minima ANI to G16 RMSD ≤ 0.5 Å
Mol1	4	5	4
Mol2	4	8	4
Mol3	4	6	6
Mol4	4	4	4
Mol5	4	6	4
Mol6	8	8	8
Mol7	5	6	5
Mol8	4	5	4

### Success of DeepConf vs Auto3D on Global/Local Minima Match to G16

#### Starting with a near Equilibrium Structure

In the next step, we assessed the DeepConf and Auto3D predicted minima with respect to G16 predicted ones starting with G16 optimized global minima for these compounds. In this part, instead of manually creating intermediate structures, we started with G16 equilibrium and used the two conformer generation algorithms to find global and local minima. We defined the following success criteria for minima generation by the two conformer algorithms. If the conformer generation algorithms suggested minima match the G16 predicted ones by means that RMSD is less than or equal to 0.5 Å, then we considered this as successful. The rationale for choosing 0.5 Å as the success threshold is that the G16-predicted conformers for these small ligands are separated by at least this RMSD value (Figure S1). So, covering all the G16 predicted minima by DeepConf (or Auto3D) within the RMSD of 0.5 Å would yield 100% success. Assigning the lowest energy structure to “global” and other minima to “local”, the success on global minima reflects one structure comparison while local minima reflect all possible local minima excluding the global one. %RMSD success rate = # of DeepConf (or Auto3D) minimum structures with RMSD ≤ 0.5 Å/total # G16 minimum structures.

Table 2 shows the success of conformer generation using either several parameter settings in DeepConf or Auto3D for the selected ligands when the G16 optimized global minimum is used as the initial structures in conformer generation process. Both DeepConf and Auto3D are adjusted to use the most similar parameter settings to assess their performance. Common and differed parameters are given in Table S1. In Auto3D, five out of eight structures have RMSD ≤ 0.5 Å of Auto3D assigned global minimum to the G16 assigned global minimum, yielding an overall success of 63%. On the other hand, when DeepConf is used, the overall success can increase up to 75%. Since DeepConf has versatility in picking different numbers of structures for each torsion point by two different options as “nfold” and “npick”, one could obtain improved results. These features are not available in Auto3D, which totally relies on RdKit and/or ETKDG to generate numbers of conformers. Although DeepConf also uses RDkit and ETKDG, we can adjust the number of rotations for each torsion (nfold) and we further cluster these structures by *K*-means clustering and pick more structures from each cluster (npick) that allows the assessment of multiple points (corresponding to more than one dihedral angle for each cluster) from each of the torsion points. With DeepConf, we also observed slight improvement on finding local minima.

**Table 2:** Success of Conformer Generation on Selected Compounds with Different Parameter Settings (Parset) When the Initial Structure is Near Equilibrium^a^

	DeepConf: parset1				DeepConf: parset2				DeepConf: parset3			
		global to G16 global		local to G16 local		global to G16 global		local to G16 local		global to G16 global		local to G16 local
ID	# conf	RMSD (Å)	Δ*E* (kcal/mol)	success	# conf	RMSD (Å)	Δ*E* (kcal/mol)	success	# conf	RMSD (Å)	Δ*E* (kcal/mol)	success
Mol1	2	0.01	0.1	33%	2	0.01	0.1	33%	2	0.01	0.1	33%
Mol2	11	0.71	–0.1	67%	3	0.17	–1.9	33%	8	0.17	–1.9	67%
Mol3	3	0.85	0.0	33%	2	0.86	0.8	33%	3	0.86	0.8	33%
Mol4	2	0.01	0.0	33%	2	0.02	–0.2	33%	2	0.02	–0.2	33%
Mol5	2	0.81	0.0	0%	2	0.80	0.0	0%	2	0.80	0.0	0%
Mol6	7	0.05	0.0	43%	2	0.09	0.2	14%	6	0.09	0.2	43%
Mol7	5	0.61	0.0	50%	3	0.35	–0.1	25%	5	0.66	–0.3	50%
Mol8	3	0.01	–0.1	33%	2	0.01	–1.3	33%	4	0.01	–1.3	67%
overall		50%	0.0	37%		75%	0.6	26%		63%	0.6	41%

	DeepConf: parset4				DeepConf: parset5				Auto3D			
		global to G16 global		local to G16 local		global to G16 global		local to G16 local		Auto3D global to G16 global		Auto3D local to G16 local
ID	# conf	RMSD (Å)	Δ*E* (kcal/mol)	success	# conf	RMSD (Å)	Δ*E* (kcal/mol)	success	# conf	RMSD (Å)	Δ*E* (kcal/mol)	success
Mol1	2	0.01	0.2	33%	2	0.03	0.2	33%	1	0.01	0.0	0%
Mol2	29	0.20	–1.8	100%	11	0.25	–1.7	100%	8	0.17	–2.1	67%
Mol3	4	0.86	0.8	33%	3	0.86	0.7	33%	2	0.86	0.6	33%
Mol4	2	0.01	–0.2	33%	2	0.02	–0.2	33%	2	0.02	–0.1	33%
Mol5	2	0.79	0.1	0%	2	0.05	0.1	0%	3	0.05	–0.1	33%
Mol6	10	0.10	0.2	71%	7	0.55	0.2	43%	10	0.55	0.2	43%
Mol7	6	0.61	–1.0	75%	6	0.02	–1.0	50%	5	0.61	–1.1	50%
Mol8	3	0.01	–1.3	33%	3	0.01	–1.3	33%	3	0.05	–1.3	33%
overall		63%	0.7	47%		75%	0.7	41%		63%	0.7	37%

a For the details of parsets, refer to Table S1.

Table 2 also reports the Δ*E* values of DeepConf and Auto3D relative to G16-calculated energies for the global minima. The data suggests that the energies of global minima are accurately reproduced by both algorithms. Small negative or positive deviations fall within the precision margins of the ANI-ML potentials and may also result from slight structural shifts. To validate this, we recalculated energies using G16 at the equilibrium geometries produced by Auto3D and DeepConf. These deviations should not be misinterpreted as ML potentials identifying lower-energy structures. This does not impact the success of the conformer generation, which is based on RMSD rather than energies.

Among all the parsets we tested, we conclude that parset 2 is best for computational efficiency with slightly reduced accuracy, parset 4 is best for comprehensively covering both local and global minima at a higher computational cost, and parset 5 is the optimal choice for a balance between accuracy and computational cost (Tables S1 and S5).

One should note that when G16 is used in optimization process, it considers four different criteria to converge (i.e., maximum force, RMS force, maximum distance, and RMS distance) whereas DeepConf and Auto3D considers only energy with respect to coordinate (i.e., maximum force) to converge.

#### Starting with Off-Equilibrium Structure

To evaluate the optimization processes for DeepConf, the G16-equilibrated test molecules in Table 2 were distorted using two different approaches (“distortion 1” and “distortion 2”) to generate 100 off-equilibrium geometries each. In the first approach (“distortion 1”), directly the *x*,*y*,*z* coordinates were randomly changed so as to produce structures that have RMSD to the initial (G16 equilibrated) structure between 0.5 Å (i.e., minimum RMSD) and 2.0 Å (i.e., maximum RMSD). In the second approach, the structures are placed in a box and the box is uniformly scaled between 0.96 and 1.10 Å. Thus, the molecule is contracted or expanded uniformly. Finally, the coordinates are randomly scaled between 0.5 and 1.5 to produce off-equilibrium geometries (“distortion 2”). QM optimizations confirmed that all distorted geometries converged to either global or local minima.

Table 3 compares how effectively DeepConf recovers low-lying structures from these distorted inputs. DeepConf directly optimizes from coordinates and successfully recovers the nearest local or global minimum in most cases (98% for “distortion 1” and 60% for “distortion 2”). We should also note that Auto3D is not designed for direct geometry optimization rather it produces the lowest energy conformer no matter what starting structure is given even though the parameters are set to yield 1 conformer only. On the other hand, DeepConf is designed to overcome such issues by attempting to optimize directly from coordinates.

**Table 3:** Optimization Performance of DeepConf When Initial Structures are beyond Equilibrium Geometry^a^

	DeepConf	
ID	distortion 1	distortion 2
Mol1	99.0%	51.0%
Mol2	98.0%	53.0%
Mol3	99.0%	63.0%
Mol4	98.0%	56.0%
Mol5	96.0%	67.0%
Mol6	99.0%	71.0%
Mol7	97.0%	69.0%
Mol8	97.0%	50.0%
overall	98%	60%

a The success shows the number of instances where optimization is converged to a geometry with RMSD ≤ 0.5 Å.

In addition to the previously described distortion methods (“distortion 1” and “distortion 2”), we introduced a third distortion approach (“distortion 3”) to further evaluate the optimization processes for DeepConf. Distortion 3 was generated by performing 1 ns classical MD simulations in vacuum at an elevated temperature of 1000 K using GAFF parameters and RESP charges in an *NVT* ensemble. From each trajectory, 100 frames were extracted and subjected to optimizations using QM and DeepConf. All of these structures converged to either global or local minima during QM optimization. As expected, most MD-sampled conformations remained close to equilibrium, restrained by the harmonic nature of classical MD simulations. Consequently, most of them are also converged to one of the minima by DeepConf. However, there were still several instances where DeepConf optimizations did not match any of the known minima within the RMSD threshold. These mismatches likely reflect the challenges posed by nonsmooth regions of the PES. Detailed comparisons of these results, including the distributions of recovered minima, are provided in the Supporting Information (Table S2).

We also tested the performance of different optimization algorithms implemented in our script for navigating nonsmooth regions of the PES. As a case study, we optimized the H–F bond starting from an initial separation of 3.0 Å using multiple minimization methods. For such a simple system, the computational cost was negligible, and all optimizations completed within seconds. However, we observed that LBGS and FIRE often became stuck in the nonsmooth region, yielding invalid geometries upon optimization. The success of these methods depended strongly on the initial distance. Conversely, BFGS and GPMin successfully converged to the equilibrium geometry across a range of starting distances. When applied to the selected set of 8 compounds, all four algorithms produced similar results in terms of equilibrium structures. GPMin was the most successful in navigating nonsmooth regions but was significantly impacted by the number of optimization steps, with its performance slowing exponentially as the step count increased. BFGS also showed strong performance but failed to converge for a non-negligible number of initial geometries. While a detailed analysis of these observations is beyond the scope of this study, we believe FIRE and BFGS represent the best trade-offs between computational cost and convergence for most systems.

### Finding Bioactive Conformations with DeepConf and Auto3D

One of the ultimate goals in drug discovery and design is to computationally screen many drug candidates to the biological target molecules and is to find the most plausible binding mode as well as binding strength using different strategies such as molecular docking, MD simulations and free energy calculations. For instance, in molecular docking both search algorithms and scoring functions are limited to the attempts the software makes during the docking process. Hence, the conformer generation prior to docking can improve both search and scoring efficiency by creating a good starting structure. For this reason, most conformer generation algorithms are assessed for their performance on finding the bioactive conformations.

In this step, we assess the performance of Auto3D and DeepConf to reproduce the bioactive conformations in the absence of target biomolecule. We have followed a unique protocol which can be a guideline for benchmarking conformer generation algorithms’ performance to capture the bioactive conformation.

**Figure 5:**
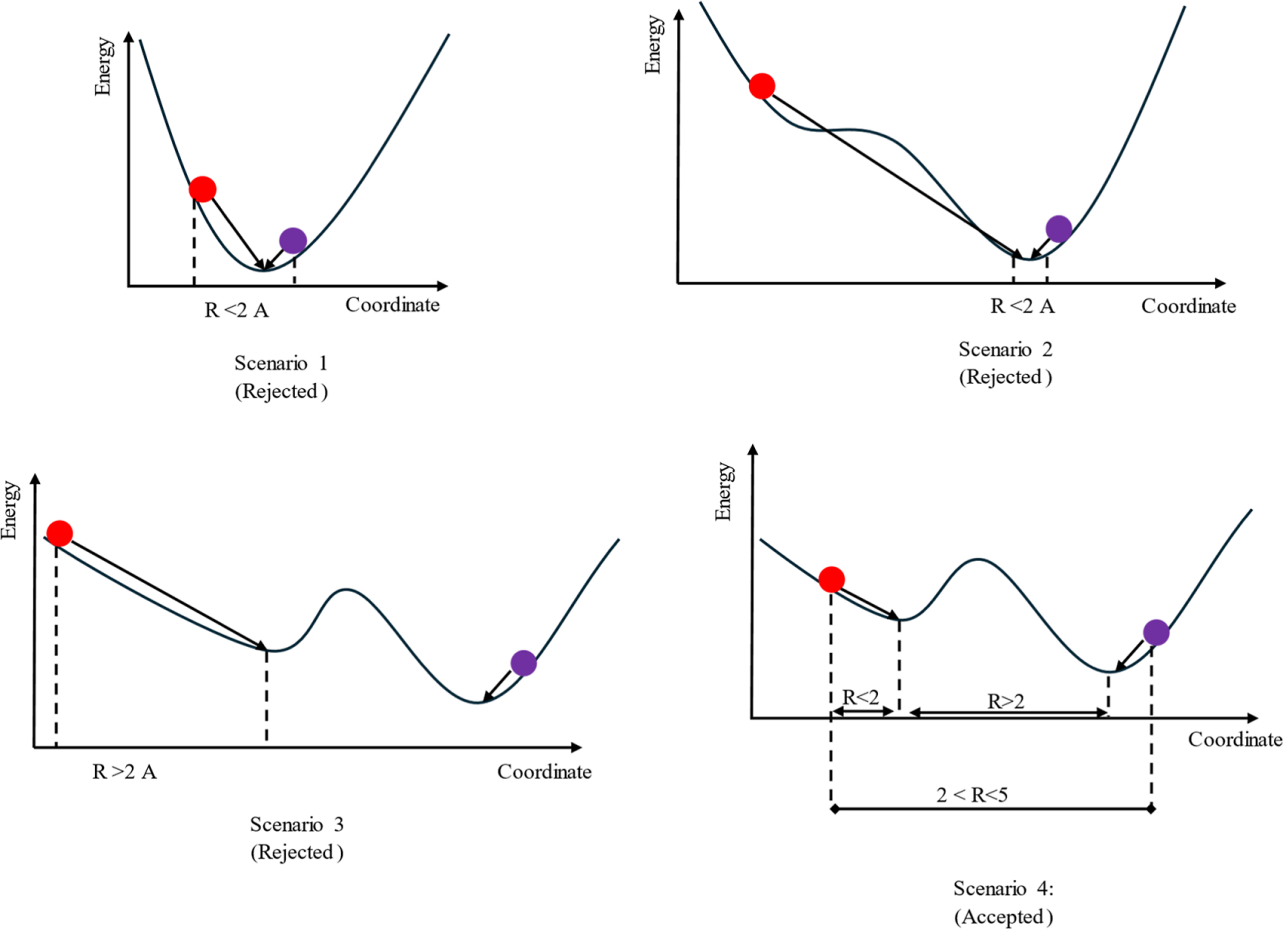
Schematic representation of performance of conformer generation algorithms for capturing bioactive conformation. The blue and red balls represent the ideal and model structures, respectively. Solid (one-sided) arrows show the structural change upon a QM optimization.

The Protein Data Bank (PDB) reports “model” structures as the ligand conformations found in the presence of biomacromolecules such as proteins (i.e., bioactive conformations). PDB also reports “ideal” structures to represent a chemically reasonable, idealized structure of the ligand that is optimized to ensure correct bond lengths, bond angles, and stereochemistry. The “ideal” structure is not explicitly meant to be the global minimum; its purpose is to serve as a high-quality, chemically valid model for applications such as docking, visualization, and further computational studies. Our protocol begins with the idea that the “model” and “ideal” structures correspond to two different energetically low-lying structures. Thus, after ensuring “ideal” and “model” structures for each ligand that represent different conformations, starting with an “ideal” structure (one of the minima) and finding “model” structure (another of the minima) with conformer generation process would indicate the success of the algorithm. To assess the true performance of a conformer generation algorithm, we have considered the following scenarios and eliminated most structures according to the criteria defined in each scenario (Figure 5).

Scenario 1: The “ideal” and “model” structures of a ligand may correspond to the same conformation (minimum). If this is the case, QM optimization of “ideal” structure will already yield to the same structure with QM optimized “model” structure. Including these structures in conformer generation process would bring additional (unrealistic) success. Thus, we have excluded such cases by comparing the RMSD of heavy atoms of “ideal” and “model” structures. We rejected structures with RMSD ≤ 2 Å (ideal to model). By applying this criterion, we have also avoided the cumbersome QM optimization of thousands of ligands.

Scenario 2: ideal and model structures may correspond to different conformers but the energy barrier connecting them might be too small. In this case, like scenario 1, QM optimization of “ideal” structure would still yield the same conformer as QM optimized “model” structure. We have compared the optimized structures of “ideal” and “model” structures and rejected those with RMSD ≤ 2 Å (ideal to model).

Scenario 3: although ideal and model structures may correspond to different conformers, the model structure might be too far from its local minimum. If this is the case, assumedly the ligand’s bioactive conformation corresponds to a structure that is mostly affected by nearby atoms (of the biomacromolecule) and the ligand has a strain energy to a considerable extent. Since we optimized the model structure in the absence of the biomacromolecule, the ligand relaxes to the closest unstrained conformation, which is too far from the bioactive conformation. Optimization of model structure would change the geometry a lot. Since it is not near a minimum, no optimization algorithm would find these structures. Therefore, we excluded these structures by only accepting RMSD ≤ 2 Å of QM optimized “model” against initial (unoptimized) “model” structures. This also avoids miscalculations of the performance by RMSD for the conformer generation since the unoptimized and QM optimized bioactive conformations differ drastically.

Scenario 4: This is the case we have assessed the performance of conformer generation (accepted scenario). In this case, the model and ideal initial structures are at least 2 Å apart in terms of RMSD; optimization of both structures still converges to different minima; and the initial model structure already holds near minimum geometry.

We believe that this protocol outlined here should give the true performance of any conformer generation algorithm when assessed for bioactive conformation capturing when started from ideal structures of the ligands in the absence of the target biomacromolecule.

We have downloaded 48,485 structures, and among these only 43,357 ligands had both model.sdf and ideal.sdf structures available in the database. To ease the computation, we have only included the structures that have the number of heavy atoms in the range of 15–30 and that have torsion points that are in the range of 2–5. We have also eliminated structures that have other atoms than C, H, N, O, F and Cl in their structure since ANI-ML potentials can only manage these atoms (i.e., NNP is trained only for these atoms). Only 34,107 ligands have passed from these filters. Among these, 762 ligands had the 2 Å ≤ RMSD ≤ 5 Å values of initial “model” and “ideal” structures. Normally, there is no upper limit to RMSD values, and one could choose all that are above 2.0 Å (i.e., scenario 1 is applied). However, our choice of 5 Å maximum is to optimize structures at the DFT level in a feasible time. We have optimized these 762 ligands at the WB97X/6-31G* level and recompared the RMSD values (between model and ideal structures) upon optimization. Only 439 structures with RMSD ≥ 2 Å between model and ideal (i.e., scenario 2 is applied) and that RMSD ≤ 2 Å between optimized model and unoptimized model structures (i.e., scenario 3 is applied) have remained after this step. These structures are assured to hold different “model” and “ideal” QM optimized conformations. Our aim for assessing the performance of the conformer generation is the ability to find the QM optimized “model” structure when QM optimized “ideal” structure is introduced to the program among these final 439 selected structures (Table S3).

**Figure 6:**
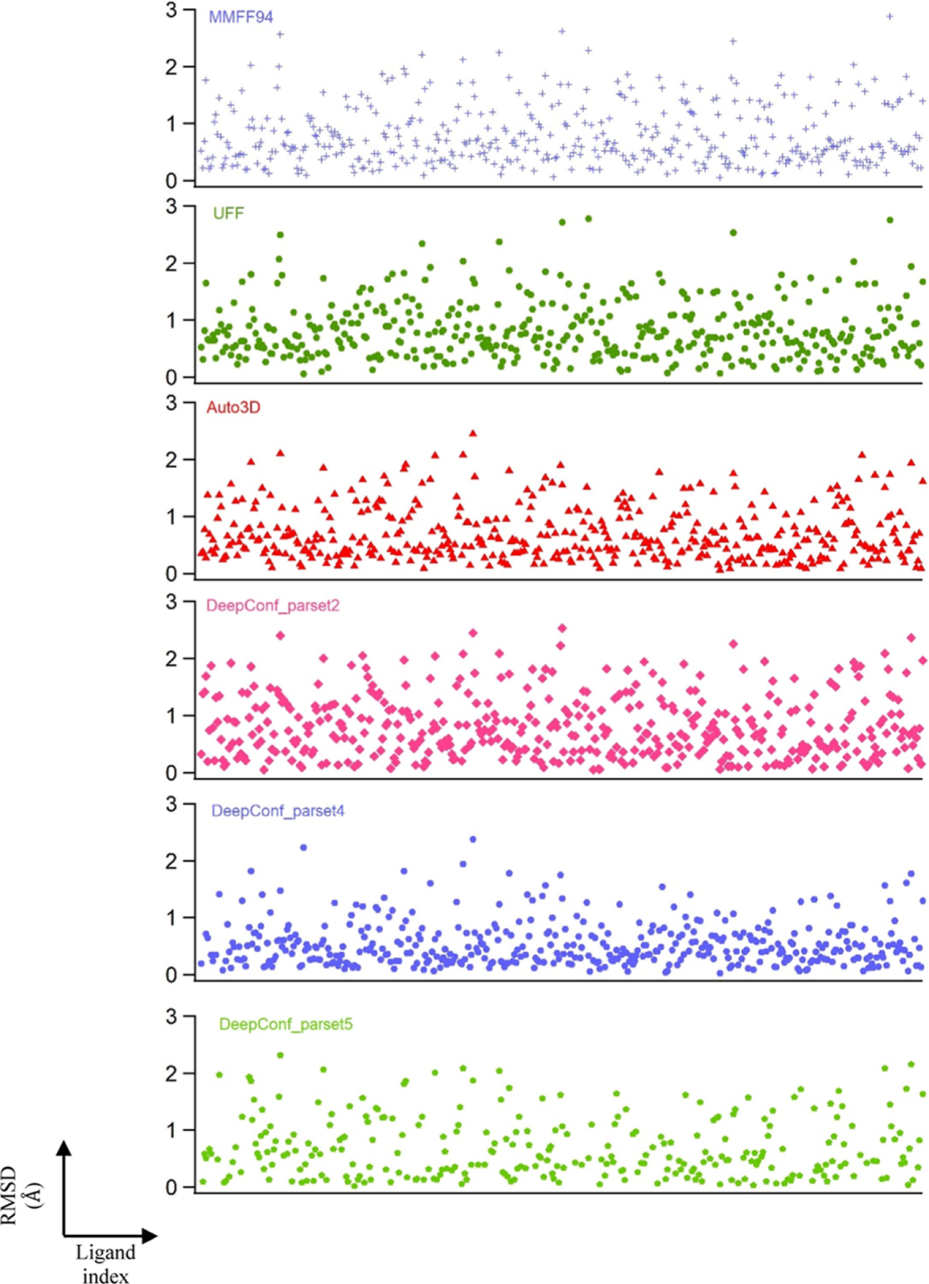
Distribution of minimum RMSD values of all conformers generated by different algorithms.

Table 4 shows the summary of the performance of both DeepConf and Auto3D along with conventional conformer generation by RDKit using two different classical force fields to reproduce the model structures starting from ideal structures. Auto3D has produced an average of 62 conformers per ligand. In the table, the “top” refers to the lowest energy structure. We assigned as “successful” when at least one of the generated conformers considering the top 1, 3, 10 and all conformers are within the threshold of an RMSD of 0.5 Å (Table 4 and Figure 6) along with the cutoffs of 1.0, 1.5, and 2.0 Å (Table S3). For Auto3D, when all the conformers are considered, the success of at least one conformer to be within 0.5 Å RMSD from the model structure when the initial structure is given as the ideal structure is 43.5% with an average ensemble size of 62. This value is 28% when only top 10 conformers are considered. We also analyzed the coverage of QM optimized “model” and “ideal” structures within the top 3 and 10 (i.e., the 3 or 10 lowest energy) structures predicted by the conformer generations. With the RMSD cutoffs from 0.5 to 2 Å, the success ranges from 15.3 to 18.5% to 78.1–84.7% in model and ideal structures. Thus, if one would produce only three lowest energy structures out from Auto3D, the success of covering both model and ideal structure within 0.5 Å of RMSD cutoff is 15.3–18.5%. In the consideration of the top 10 conformers, these numbers reach 27.3–39.4%.

**Table 4:** Overall Performance of DeepConf and Auto3D in Reproduction of Model/Ideal Structures from Ideal Structures^b^

				wrt/QM optimized model				wrt/QM optimized ideal				cumulative (model + ideal)
Method	Parameter	RMSD	size	success %	mean	min	max	success %	mean	min	max	success %
DeepConf	parset2	top 1		9.8				9.6				9.7
		top 3		21.6	1.25	0.06	3.59	20.3	1.21	0.03	3.13	21.0
		top 10		31.7	0.92	0.06	2.89	43.1	0.70	0.03	2.47	37.4
		all	18	35.5	0.81	0.06	2.53	54.7	0.54	0.03	2.24	45.1
	parset4	top 1		10.3				5.8				8.1
		top 3		20.6	1.31	0.03	3.92	12.9	1.46	0.06	3.37	16.7
		top 10		32.7	1.01	0.03	3.92	25.9	1.04	0.04	3.09	29.3
		all	322	56.5	0.54	0.03	2.38	71.0	0.39	0.02	2.01	63.8
	parset5^a^	top 1		13.4				7.4				23.7
		top 3		23.0	1.23	0.05	3.96	18.7	1.28	0.04	3.18	22.7
		top 10		34.6	0.93	0.04	3.00	40.6	0.72	0.04	2.34	37.1
		all	36	46.6	0.69	0.02	2.32	67.5	0.43	0.03	1.59	56.2
	parset6	top 1		4.8				8.4				6.6
		top 3		9.3	1.51	0.12	3.93	19.6	1.06	0.12	3.08	14.5
		top 10		21.0	1.09	0.12	3.10	33.3	0.76	0.08	2.54	27.1
		all	21	23.5	0.93	0.10	3.10	36.7	0.66	0.11	2.35	30.1
	parset7	top 1		4.8				8.4				6.6
		top 3		9.1	1.57	0.08	3.85	17.1	1.15	0.10	3.10	13.1
		top 10		18.0	1.19	0.08	3.56	33.0	0.82	0.06	2.52	25.5
		all	207	37.1	0.71	0.07	2.27	58.8	0.49	0.06	2.00	47.9
Auto3D	Auto3D	top 1		7.5				7.5				7.5
		top 3		15.0	1.36	0.06	4.11	18.2	1.24	0.03	3.39	16.6
		top 10		28.2	0.98	0.06	3.66	40.5	0.78	0.03	2.66	34.4
		all	62	43.5	0.69	0.06	2.45	65.4	0.47	0.03	1.80	54.4
RDKit	MMFF94	top 1		6.8				5.9				6.4
		top 3		15.5	1.33	0.05	3.36	16.4	1.28	0.05	3.38	15.9
		top 10		29.6	0.95	0.05	3.14	37.8	0.76	0.05	2.58	33.7
		all	20	35.8	0.78	0.05	2.88	47.8	0.59	0.05	2.05	41.8
	UFF	top 1		8.0				5.2				6.6
		top 3		14.8	1.27	0.06	3.58	13.9	1.39	0.05	3.41	14.3
		top 10		24.6	0.93	0.06	3.07	30.3	0.79	0.05	2.50	27.4
		all	20	29.4	0.82	0.06	2.78	38.7	0.65	0.05	2.42	34.1

a Subset of 282 compounds.

b The number values in RMSD columns show the success percentage (%) with the corresponding criteria.

When the same analysis is performed by DeepConf with different parameter settings (parsets), we produced somewhat comparable results to Auto3D in fewer conformer (ensemble) sizes. The average number of conformers generated by parset2 was as few as 18. The success of capturing model and ideal structures within the top three/ten structures was slightly greater than Auto3D. In parset5, the success can increase up to 40.6% and 67.5% considering top 10 and all ensembles, respectively within only an average size of 32 conformers.

When a similar analysis using RDKit using MMFF94 was performed, 29.6% success was observed when considering the top 10 conformers with respect to QM optimized model structures. RDKit with UFF force field was even lower than that with 24.6% success. In most cases, Auto3D and our DeepConf have surpassed the conformations generated by conventional methods.

In addition, we also generated the conformers via DeepConf without any optimization and picked the minimum energy structure among these conformers. This way of getting conformers is much faster since no optimization is involved, and only single point energies (SPE) are used in ranking the conformers. Parset6 and parset7 use the same values as parset2 with the only difference of no optimization (i.e., SPE only) in both and clustering is also off in the latter. These two settings show quite successful performance with respect to their tremendously low computational cost.

Apart from our definition of success rates, we also compared the results in analogy to earlier studies, which reported the mean, minimum and maximum RMSD values without any success conditions by means of cutoff in RMSD. Friedrich et al.^52^ have reported benchmarking of numerous conformer generation algorithms using PLATINUM diverse data set^59^ (for ∼2859 molecules) and observed mean RMSD of 0.79 Å for RDKit using MMFF94 and 0.82 using UFF when maximum ensemble size is set to 50. These results are in perfect agreement with our predictions of 0.78 and 0.82 Å (in the ensemble sizes of 20), respectively, for RDKit using MMFF94 and UFF for prediction of QM optimized model structures starting from QM optimized ideal structures. In the same study, the best performance among 22 different algorithms (of ConfGenX by Schrodinger) were found to have the mean (of the closest structures) RMSD of 0.63 and 0.54 Å for maximum cluster sizes of 50 and 250, respectively. In another study,^59^ they have also reported that RDKit gives the performance of 0.98 Å when the cluster size is 10, which is also in perfect agreement with our finding, which is 0.93–0.95 Å for the top 10 structure evaluation. The lowest value they could get was 0.59 Å when they generated up to 500 conformers using the same method. One of the most recent studies by Seidel et al.^4^ reported that RDKit utilized with ETKDGv3 in the same force fields (MMFF94 and UFF) has a performance of mean RMSD of 0.74 and 0.71 Å for a maximum ensemble size of 50. Their introduced method, so-called “CONFORGE”, yielded 0.67 Å for the same value. As a conclusion, to the best of our knowledge none of the studies could bring this value below 0.5 Å no matter what maximum ensemble size is used.

When the same performance analysis was performed for Auto3D, we observed the mean RMSD was as low as 0.69 Å for model structures and 0.47 Å (Table 4) for ideal structures in an average ensemble size of 62 (For individual ensemble sizes and RMSD values, refer to Supporting Information). Similarly, our DeepConf with different parameter settings (Table S4) could produce mean RMSD as low as 0.54 Å for model and 0.39 Å for ideal structures. Here, “parset2” uses 180° (nfold = 2) for each torsion point and produces the mean RMSD of 0.81 Å (model) and 0.54 Å (ideal) in just 18 average ensemble size. Moreover, by increasing the “nfold”, one can easily boost up the performance of DeepConf. Here, in our test of parset4, where nfold = 4 (i.e., 90° rotations on each torsion), we could improve the RMSD values to 0.54 and 0.39 Å for model and ideal structures, respectively. One might argue that the ensemble size is also increased tremendously (up to 322 in parset4). This is mostly because we have set the convergence criteria to 0.02 eV/Å (maximum force) in all parameter settings to reduce the computational cost. When lower convergence criteria applied, the ensemble size would drop to tens rather than hundreds.

We also evaluated the performance of our DeepConf in the case of no-optimization involvement and thus based on single point energy (SPE) calculations since sometimes, it might be desired to generate the conformers as fast as possible without waiting for optimization process. Parset6 and parset7 use the same values as parset2 with the only difference of no optimization (i.e., SPE only) in both and clustering is also off in the latter.

Parset5 uses similar clustering to parset2, but instead of picking the lowest energy structure from each clusters, it picks 2 additional structures. Therefore, for each of the “nfold” clusters a total of 3 structures are optimized. By doing this, mistakes by the *K*-means clustering are attempted to overcome. This improves the performance of reproducing QM optimized model and ideal structures. Within 36 total suggested local minima, we observed a success of 46.6% in model and 67.1% in ideal structures based on the RMSD ≤ 0.5 Å. Mean RMSD values of among all conformers generated are observed as 0.69 and 0.43 Å for model and ideal structures, respectively.

We also assessed the performance of our DeepConf in the case of no-optimization involvement and thus based on single point energy (SPE) calculations since sometimes, it might be desired to generate the conformers as fast as possible without waiting for optimization process. These two settings show quite successful performance with respect to their tremendously low computational cost.

With the assumption that model structures and ideal structures represent two different minima for any ligand, RMSD values of the conformers produced by DeepConf, Auto3D or RDKit against bioactive conformations are a good indicator to show the success of the conformer generation algorithms. However, this is only one part of the problem and the energy difference between model and ideal structure also needs to be distinguished by the algorithm. For this reason, we have also analyzed the results by comparing relative energies among conformers (Table 5).

**Table 5:** Performance of Conformer Generation Algorithms on Reproducing QM Optimized Energies^b^

			Δ*E*^ML-QM^ (kcal/mol)			auccess energy cutoff			
method	parameter		MUD	min	max	*kT*	2*kT*	3*kT*	count
DeepConf	parset2	model	2.65	–6.7	24.14	14.5%	35.0%	44.4%	117
		ideal	2.66	–7.44	12.2	14.5%	27.3%	38.5%	
	parset5^a^	model	2.91	–14.7	11.3	13.0%	23.0%	41.0%	100
		ideal	2.68	–7.5	12.1	17.0%	27.0%	40.0%	
Auto3D	auto3d	model	3.32	–10.1	18.5	11.0%	25.5%	37.9%	145
		ideal	2.87	–5.0	16.7	13.1%	24.8%	35.9%	

a Subset of 282 compounds.

b Each *kT* refers to 0.592 kcal/mol and MUD is the abbreviation of mean unsigned difference (in kcal/mol) over all evaluated ligands.

In this step, we have first compared the absolute energies produced by DeepConf and Auto3D to their QM optimized analogues (For, explicit energies and differences of individual ligands, refer Supporting Information). The energy differences QM optimized model and ideal structures, Δ*E*_model-ideal_^QM^, span in a range from −14.96 to 27.20 kcal/mol (Table 5). As the ideal structure is not explicitly meant to be the global minimum and our procedure for selecting ligands only assured that QM optimized model and ideal structures represent two different minima; a model structure can have lower energy than the ideal structure. Although, the relative energy between the model and the ideal structures range −14.96 to 27.20 kcal/mol, the mean energy difference, ⟨Δ*E*_model-ideal_^QM^⟩, among all ligands is just 2.75 kcal/mol, clearly indicating the energetically close structures.

QM optimized structures (model or ideal) represent a single minimum whereas conformer generation algorithms generate many other minima alongside model and ideal structures when the algorithm is initiated with QM optimized ideal structures. These conformers are already reported as ensemble size.

To calculate the mean unsigned difference (MUD) values between the conformer generation algorithm’s predicted structures and the QM-optimized structures, we used the energy values of structures with the minimum RMSD to the QM-optimized reference. This approach was applied even if the algorithm identified lower-energy structures, as we focused on conformers within an RMSD cutoff of 0.5 Å, which are assumed to represent the same conformer identified by both QM and ML methods. This is because our reference points are QM optimized model and ideal structures, and we have not thoroughly explored all the conformers with QM calculations due to computational cost. The MUD values of DeepConf in predicting both QM optimized model and ideal structures (i.e., ⟨Δ*E*_model_^ML-QM^⟩ and ⟨Δ*E*_ideal_^ML-QM^⟩), are 2.65 and 2.66 kcal/mol, respectively, with a span from −6 to 24 kcal/mol for the model structures, and in a range of −7 to 12 kcal/mol on ideal structures. Similarly, the MUD value of ideal structures in Auto3D is 2.87 kcal/mol ranging between −5 to 17 kcal/mol. The MUD values of Auto3D predicted model structures deviate from the QM optimized model structures by 3.32 kcal/mol in a range of −10 and 18 kcal/mol, indicating slightly less confidence. Unfortunately, RDKit predictions using either MMFF94 or UFF do not give absolute energy values, so we could not directly compare RDKit predicted absolute energies to QM optimized energies.

**Figure 7:**
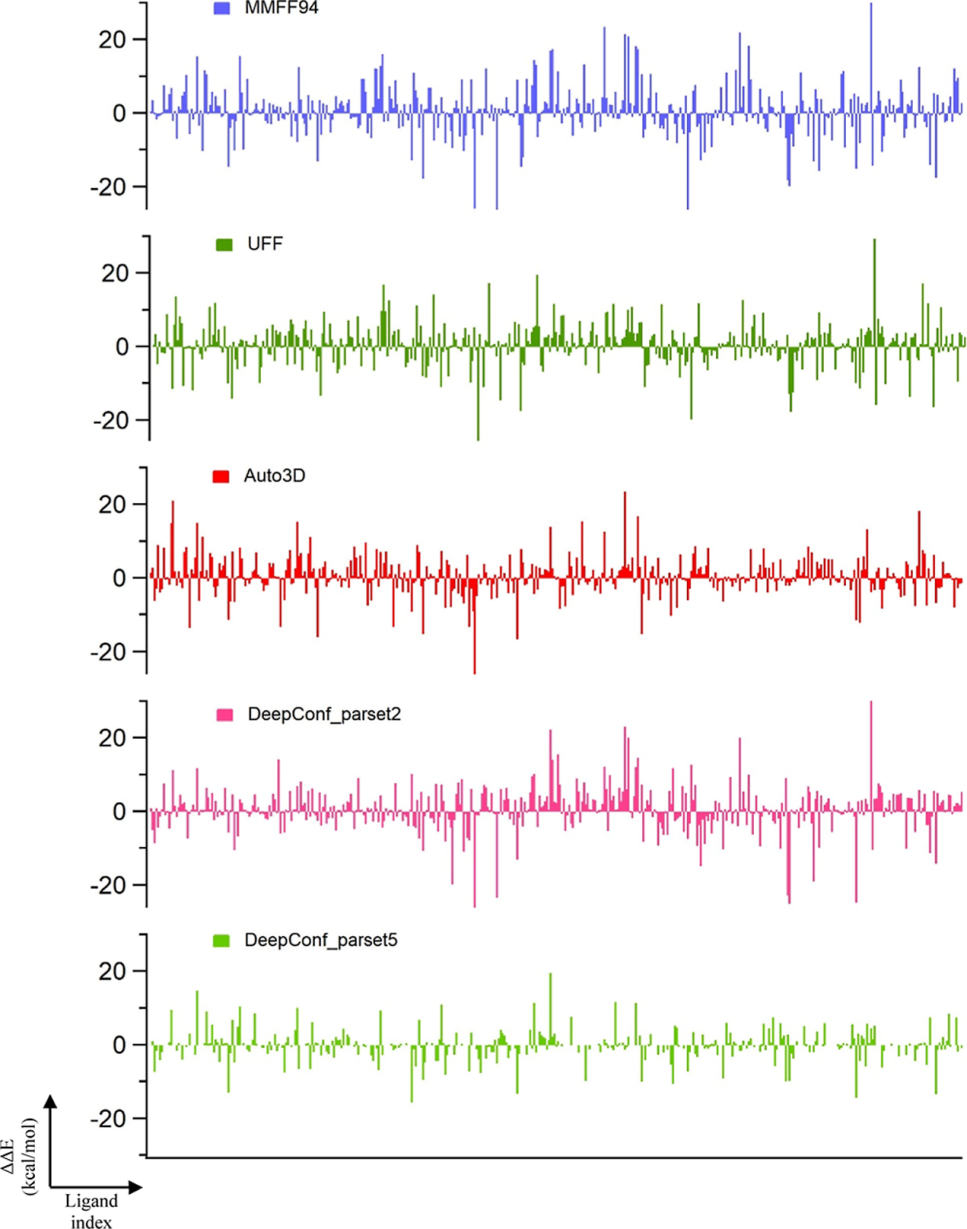
Distribution of relative energy differences (ΔΔ*E* = Δ_model-ideal_^conf^ – Δ_model-ideal_^QM^) of conformers generated by different algorithms.

We also considered how the conformer generation algorithms perform predicting the energy gap between the two minima (QM optimized model and ideal) since this gap is related to the relative populations (Table 6 and Figure 7). In this analysis, Δ*E*_model-ideal_^QM^ (i.e., the energy difference between QM optimized model and ideal structures) as the reference relative energy and Δ*E*_model-ideal_^conf^ in which conformer generation algorithms’ best solutions (the ones with minimum RMSD with ≤0.5 Å) were selected as the sample. Thus, the success on the relative energies corresponds to ΔΔ*E* = Δ_model-ideal_^conf^ – Δ_model*-*ideal_^QM^. The algorithm is assumed to be successful for a ligand when ΔΔ*E* is less than *kT* energy of 0.592 kcal/mol. It should be noted that the success of the relative energies suffers from the low performance of the RMSD values. DeepConf with parset2 finds 30.7% within *kT* energy margin between the model and ideal structures while Auto3D finds 23.4%. Interestingly, MMFF94 based RDKit predictions are also quite comparable to Auto3D especially when the margin is increased to larger *kT* values. Yet, DeepConf predicted success is larger than all the other three methods.

**Table 6:** Performance of Conformer Generation Algorithms on Reproducing Relative Energies between model and Ideal Structures^b^

		ΔΔ*E* (kcal/mol)	success energy cutoff			count
method	parameter	MUD	*kT*	2*kT*	3*kT*	
DeepConf	parset2	2.9	30.7%	44.4%	48.7%	117
	parset5^a^	1.8	26.0%	42.0%	58.0%	100
Auto3D	auto3d	2.6	23.4%	37.2%	46.9%	145
RDKit	MMFF94	3.3	22.3%	37.9%	48.5%	103
	UFF	3.0	16.5%	28.2%	43.5%	85

a Subset of 282 compounds.

b Each *kT* refers to 0.592 kcal/mol and MUD is the abbreviation of Mean Unsigned Difference (in kcal/mol) over all evaluated ligands.

In our previous evaluation, success was measured based on finding the “model” (bioactive) conformation starting from the “ideal” structure. Both Auto3D and DeepConf showed comparable performance, surpassing conventional conformer generation methods and reported literature values. However, this analysis only considered two conformers per ligand, leaving the question open as to how efficiently these algorithms identify the true “global” and other “local” minima.

To address this, we conducted an additional study on a subset of 21 randomly selected ligands from the 439 PDB ligands. We performed classical MD simulations for each ligand, extracting 100 frames per ligand and optimizing them using QM methods. After eliminating duplicate structures, we obtained QM reference conformers for each ligand. The lowest-energy structure among these was designated as the “global” minimum, while the remaining conformers were classified as local minima.

Table 7 presents the results of this expanded analysis, including the number of QM-optimized conformers per ligand and the ranking of bioactive conformations among them. Interestingly, bioactive conformers were predominantly ranked among the lowest 10 energy structures, with an average rank of 6.3 across the data set. On average, 37.1 unique conformers were generated per ligand from the QM optimizations of 100 MD frames.

**Table 7:** Performance of Conformer Generation Algorithms on Reproducing the True Global and Local Minima for Selected 21 Bioactive Compounds

			RMSD (Å) to G16 global														
	G16		DeepConf: parset2					DeepConf: parset5					Auto3D				
id	size	bioactive rank	size	top1	top3	top10	all	size	top1	top3	top10	all	size	top1	top3	top10	all
048	48	10	27	1.6	0.7	0.4	0.4	76	1.3	1.3	0.3	0.3	35	1.8	1.0	0.7	0.4
05B	43	3	3	1.6	1.6	1.6	1.6	11	1.8	1.8	1.6	1.6	13	1.0	1.0	0.7	0.7
0C7	13	1	21	0.7	0.7	0.7	0.7	71	3.2	3.1	2.3	0.9	99	0.6	0.6	0.6	0.6
0FT	70	2	16	1.8	1.8	0.8	0.8	47	2.0	1.8	1.2	0.8	227	1.2	1.2	0.4	0.4
0KQ	54	11	8	2.5	1.5	1.0	1.0	24	1.9	1.9	0.9	0.9	62	2.2	2.0	0.9	0.3
0KR	54	39	8	2.1	1.8	0.8	0.8	22	2.2	1.4	1.4	0.8	74	1.9	1.9	1.3	0.5
0VG	29	1	12	2.7	2.7	2.2	2.2	43	2.8	2.6	2.4	1.6	69	2.7	2.7	2.5	1.1
0YO	12	2	7	1.4	0.1	0.1	0.1	7	1.5	0.2	0.1	0.1	4	1.5	0.1	0.1	0.1
15 V	5	2	15	0.1	0.1	0.1	0.1	46	0.2	0.2	0.2	0.2	60	0.9	0.2	0.2	0.2
19R	33	4	27	1.4	1.3	0.9	0.9	93	1.5	1.4	0.6	0.6	124	1.2	1.2	0.3	0.3
1A2	75	11	8	1.9	1.4	1.4	1.4	23	2.5	0.7	0.7	0.7	62	2.2	0.4	0.4	0.4
1C7	58	2	14	0.8	0.8	0.8	0.8	46	2.9	1.8	0.9	0.8	118	2.1	0.7	0.2	0.2
1GB	42	2	15	1.6	1.6	1.6	1.6	47	2.1	2.1	2.1	1.6	105	2.5	2.2	2.2	1.0
1IF	18	15	15	0.9	0.6	0.6	0.6	42	0.4	0.4	0.4	0.4	43	2.6	1.0	0.6	0.6
21G	27	6	15	2.7	1.8	0.6	0.6	34	0.5	0.5	0.5	0.3	22	0.5	0.5	0.5	0.5
2M3	29	4	16	1.5	1.5	1.5	1.5	48	2.2	2.2	1.3	1.3	117	2.2	2.1	0.9	0.9
31T	50	5	14	1.8	1.7	1.0	1.0	35	3.1	1.7	1.0	0.3	65	0.5	0.1	0.1	0.1
33M	13	7	4	1.8	0.3	0.3	0.3	4	1.8	0.5	0.5	0.5	4	1.8	0.5	0.5	0.5
342	83	2	30	4.2	4.2	2.7	2.7	96	4.5	3.9	2.3	2.2	164	3.0	3.0	2.6	2.5
36Z	9	1	6	1.5	0.0	0.0	0.0	4	1.0	0.6	0.6	0.6	4	1.9	0.1	0.1	0.1
38W	14	3	22	2.7	2.3	0.6	0.6	53	1.1	1.1	0.9	0.9	26	1.1	1.1	1.1	0.6
mean	37.1	6.3	14.4	1.8	1.4	0.9	0.9	41.5	1.9	1.5	1.1	0.8	71.3	1.7	1.1	0.8	0.6
success %	RMSD≤	0.5		5	19	24	24		14	24	29	33		5	33	48	57
	RMSD≤	1.0		19	38	67	67		14	33	57	76		19	48	76	90
	RMSD≤	1.5		33	52	81	81		33	52	76	81		43	71	86	95

local to G16 local success %								
DeepConf: parset2			DeepConf: parset5			Auto3D		
top3	top10	all	top3	top10	all	top3	top10	all
67	90	44	67	50	26	67	80	37
33	33	33	67	50	45	67	60	46
33	50	24	67	40	11	67	40	7
0	20	19	33	40	21	0	10	17
67	38	38	67	90	42	33	70	40
33	38	38	67	50	32	67	70	35
33	20	17	33	30	7	67	50	14
67	43	43	67	43	43	33	25	25
0	10	7	0	0	4	0	0	5
67	50	19	67	70	13	33	80	12
0	13	13	33	20	9	33	40	15
67	40	29	67	70	20	67	70	20
67	40	27	33	40	15	67	40	10
0	0	0	0	0	0	0	0	2
67	50	33	67	70	35	67	70	41
67	20	13	67	20	4	0	30	6
67	40	29	33	30	26	33	30	23
33	25	25	33	25	25	0	0	0
33	30	10	33	10	3	33	20	5
0	0	0	0	0	0	0	0	0
33	50	23	33	50	13	67	50	19
40	33	23	44	38	19	38	40	18

Following the methodology of Tables 2 and 4, we analyzed the ability of DeepConf and Auto3D to predict the global and local minima among these ligands. When only the lowest-energy structure of each algorithm is considered (top1), Auto3D and DeepConf (parset2) identify the QM global minimum with 5% success, while DeepConf (parset5) achieves 14% success. When the top3, top10, or all available conformers are considered, Auto3D reaches 57% success, whereas DeepConf (parset5) reaches 33% success. The mean RMSD values for the top1, top3, and top10 conformers are similar for both methods. However, when all conformers are considered, Auto3D achieves a lower mean RMSD (0.6 Å), surpassing DeepConf. The local-to-G16 local success rates for conformers generated by DeepConf and Auto3D were also evaluated. The ensemble sizes for Auto3D, parset2, and parset5 are 71.3, 14.4, and 41.5, respectively. 38% of Auto3D’s top 3 conformers match local minima in the QM data set, while parset5 achieves 44% success in this regard.

We also investigated the effect of energy minimization steps and convergence thresholds (*f*_max_) on both Auto3D and DeepConf (Table S5). While reducing *f*_max_ lowered the ensemble sizes, it did not improve success rates or RMSD values. In fact, overly stringent minimization criteria often worsened results due to the reduction in the number of explored conformers.

## Conclusions

In conclusion, we introduce DeepConf, a novel low-energy conformer generation algorithm that utilizes ANI-ML potentials at DFT-level accuracy for efficiently reproducing bioactive conformations. Our method demonstrates robustness in challenging scenarios, such as when the initial structure is far from equilibrium or when fewer, high-quality conformers are required. By focusing on single-bond rotations, we provide the first comprehensive assessment of ANI-ML potentials using DeepConf alongside Auto3D for bioactive conformation generation. Additionally, we propose new evaluation methods that can serve as a guideline for future conformer generation algorithms. Both DeepConf and Auto3D based on ANI-ML potentials achieve a mean RMSD of less than 0.5 Å, outperforming conventional approaches. With advanced features like geometry optimization, single-point energy-based conformer generation, and compatibility with various ML potentials, DeepConf offers a powerful tool for bioactive conformation evaluation. We should also emphasize that ANI-ML potentials have an intrinsic error margin of ∼1.5 kcal/mol, which affects both DeepConf and Auto3D. This error contributes to challenges in correctly ranking global and local minima, particularly in cases where multiple low-energy conformers exist.

## Data Availability

The ANI-ML based conformer generation “DeepConf” method with tutorials and descriptions can be accessed via: [https://github.com/otayfuroglu/DeepConf]. The data set used throughout the manuscript can be accessed via: 10.5281/zenodo.14908196.

## Supporting Information Available

Supplementary figures and tables, and raw data of all outputs (PDF)
Results (XLSX)
