# Supplementary material for: DeepConf: Leveraging ANI-ML Potentials for Exploring Local Minima with Application to Bioactive Conformations
Source: J Chem Inf Model. 2025 Mar 4;65(6):2818–33. doi: 10.1021/acs.jcim.4c02053 (PMC11938341; doi:10.1021/acs.jcim.4c02053)
Supplement: Supplementary file 1 — ci4c02053_si_001.pdf [file ci4c02053_si_001.pdf]

DeepConf: Leveraging ANI-ML Potentials for Exploring Local Minima with Application to Bioactive Conformations

*Omer Tayfuroglu, Irem N. Zengin, M. Serdar Koca, and Abdulkadir Kocak**

Department of Chemistry, Gebze Technical University, 41400, Kocaeli/Turkey

* Corresponding Author: Abdulkadir Kocak, kocak@gtu.edu.tr

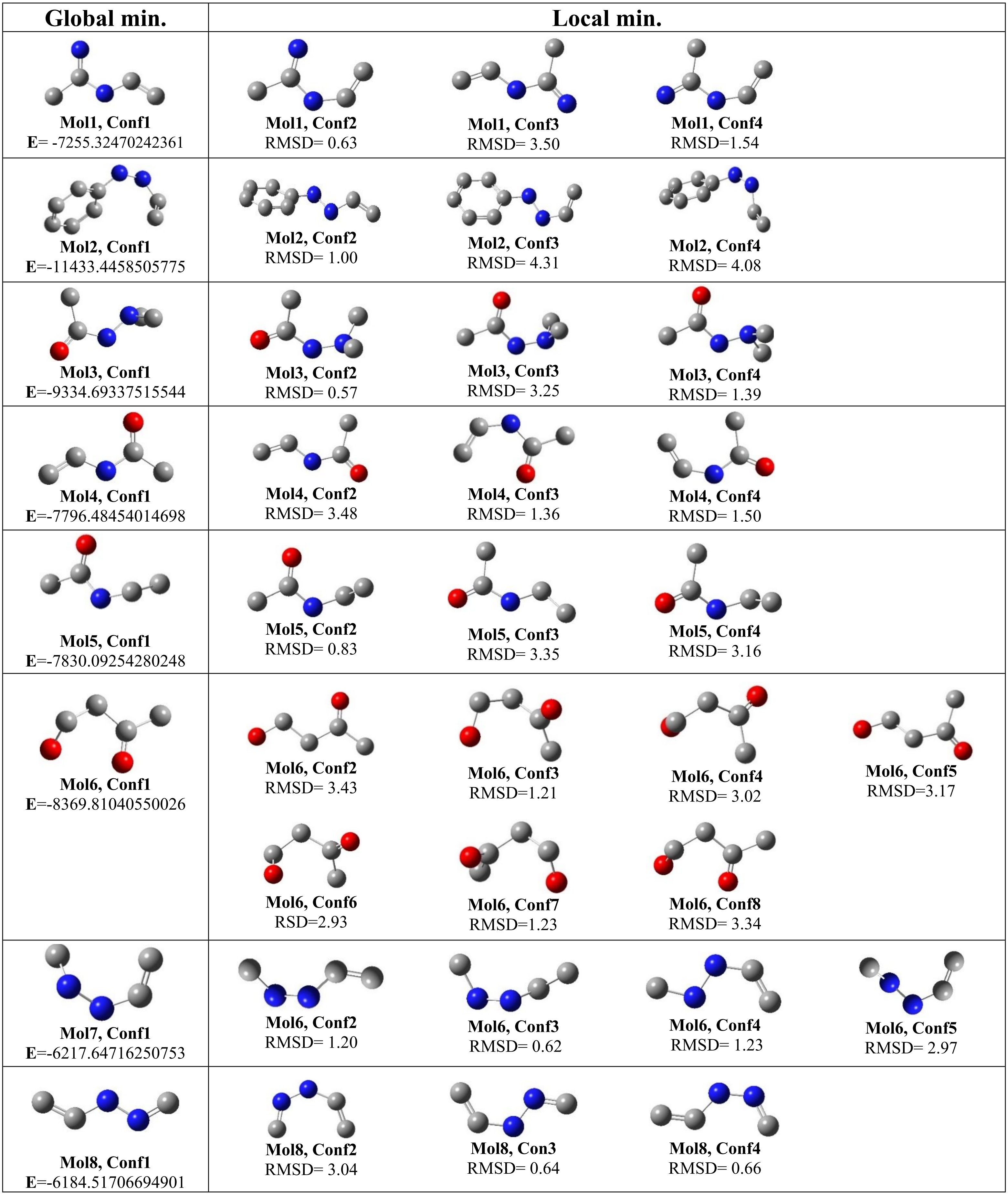

Figure S1. Initial test compounds and their G16 predicted global and local minima.

Table S1. Parameter settings used in conformer generation for the initial dataset of selected ligands. Both programs are adjusted to similar settings in order to assess their performance.

**ConfGen**	Parset1	Parset2	Parset3	Parset4	Parset5	Parset6	Parset7	**Auto3D**	Parset1
**optimization_method**	LBFGS	LBFGS	LBFGS	FIRE	FIRE	FIRE	FIRE	**k:**	FALSE
**pre_optimization_lig**	no	no	no	no	no	no	no	**window:**	100000
**genconformer**	yes	yes	yes	yes	yes	yes	yes	**memory:**	None
**ETKDG**	yes	yes	yes	yes	yes	yes	yes	**capacity:**	40
**num_conformers**	1	1	1	1	1	1	1	**enumerate_tautomer:**	FALSE
**max_attempts**	100000	100000	100000	100000	100000	100000	100000	**tauto_engine:**	rdkit'
**prune_rms_thresh**	0.5	0.5	0.5	0.5	0.5	0.5	0.5	**pKaNorm:**	FALSE
**opt_prune_rms_thresh**	0.3	0.3	0.3	0.3	0.3	0.3	0.3	**isomer_engine:**	rdkit
**opt_prune_diffE_thresh**	0.001	0.001	0.001	0.001	0.00001	0.001	0.001	**max_confs:**	None
**caculator_type**	ani2x	ani2x	ani2x	ani2x	ani2x	ani2x	ani2x	**enumerate_isomer:**	TRUE
**optimization_conf**	yes	yes	yes	yes	yes	no	no	**mode_oe:**	classic'
**optimization_lig**	no	no	no	no	no	no	no	**optimizing_engine:**	ANI2x'
**thr_fmax**	0.02	0.02	0.02	0.02	0.0002	0.02	0.02	**opt_steps:**	5000
**maxiter**	50000	50000	50000	50000	50000	50000	50000	**convergence_threshold:**	0.02
**nfold**	4	2	2	4	2	2	2	**patience:**	1000
**npick**	0	0	2	0	2	0	0	**threshold:**	0.3
**cluster**	yes	yes	yes	yes	yes	yes	no		

Table S2. Optimization of 100 frames extracted from MD simulations for the selected compounds. Matched refers to the number of structures with RMSD ≤0.5 Å to any of the minima on Figure S1. Global is the global minimum and min1,min2,etc refer to the local minima. Corresponding values under the columns show the number of structures yielded (upon optimization) to that specific minimum. Unmatched refers the structure is converged to a different minima (likely saddle point or non-smooth region).

**G16**										
**ID**	Matched/ Converged	Global	min1	min2	min3	min4	min5	min6	min7	unmatched
**Mol1**	100/100	26	2	66	6					0
**Mol2**	100/100	9	17	13	25	36				0
**Mol3**	100/100	57	5	10	28					0
**Mol4**	100/100	94	0	6	0					0
**Mol5**	98/98	29	35	17	10	7				0
**Mol6**	97/99	17	3	2	11	17	35	9	3	2
**Mol7**	100/100	7	28	36	3	10	16			0
**Mol8**	100/100	71	2	10	17					0
**DeepConf**										
**Mol1**	100/100	26	2	66	6					0
**Mol2**	81/100	4	33	19	25	0				19
**Mol3**	100/100	60	2	14	24					0
**Mol4**	100/100	94	0	6	0					0
**Mol5**	89/100	26	28	17	10	8				11
**Mol6**	95/100	16	7	2	11	19	30	7	3	5
**Mol7**	100/100	7	34	33	1	14	11			0
**Mol8**	100/100	71	3	10	16					0

Table S3. Selected (using the criteria discussed in the manuscript) 439 compounds for bioactive conformation using the conformer algorithms. Three-digit PDB codes of the ligands are followed by "M". Both model and ideal structures were optimized at WB97X/6-31G(d) level.

	QM optimized absolute energy (eV)		model to ideal			QM optimized absolute energy (eV)		model to ideal	
PDB id (M###)	model	ideal	ΔE(kcal)	RMSD (Å)	PDB id (M###)	model	ideal	ΔE(kcal)	RMSD (Å)
M048	-35013.06119	-35013.0377	-0.5416	2.35	MHDU	-24221.40445	-24221.03792	-8.4523	2.29
M05B	-36738.45331	-36738.24223	-4.8675	3.11	MHFY	-47580.09495	-47579.78741	-7.092	2.27
M06V	-33751.56273	-33751.83343	6.24261	3.25	MHGF	-35232.26863	-35232.2856	0.39136	2.77
M0BZ	-30604.61273	-30604.60568	-0.1627	2.05	MHM2	-37419.27366	-37419.25305	-0.4753	2.24
M0C5	-30414.31157	-30414.29825	-0.3071	2.08	MHNY	-24402.11204	-24402.34454	5.36145	2.11
M0C6	-43110.31197	-43110.30151	-0.2412	2.04	MHRV	-64850.06147	-64850.08707	0.59044	2.68
M0C7	-42274.12343	-42274.11433	-0.2099	2.32	MHWZ	-29060.00578	-29059.87335	-3.0538	2.3
M0FT	-33481.21534	-33480.93328	-6.5045	2.39	MHZ8	-26550.62244	-26550.57071	-1.1928	2
M0KQ	-39465.13357	-39465.15389	0.46854	2.35	MHZW	-43279.83443	-43279.9272	2.1391	1.96
M0KR	-30100.47237	-30100.47458	0.05092	2.19	MI2Q	-38907.30797	-38907.30222	-0.1327	2.23
M0LC	-37020.99388	-37020.93004	-1.4723	2.39	MI39	-32428.82236	-32428.50128	-7.4043	2.38
M0VG	-32104.53473	-32104.12248	-9.5066	2.07	MIB1	-35375.92911	-35375.88573	-1.0004	2.03
M0XF	-31543.90412	-31543.25533	-14.961	2.1	MIC8	-35592.95286	-35592.955	0.04937	2.12
M0YO	-33589.85976	-33589.77373	-1.9838	2.03	MIHE	-32072.30519	-32072.21914	-1.9844	2.19
M15V	-41341.64028	-41341.30359	-7.7641	2.21	MIUU	-36219.38248	-36219.23618	-3.3737	2.22
M19R	-25929.10389	-25928.58968	-11.858	2.44	MIUV	-39262.82273	-39262.82215	-0.0134	3.22
M1A2	-38545.76528	-38545.79904	0.77845	2.1	MIWM	-40113.06732	-40112.94598	-2.7981	2.12
M1C7	-35254.3571	-35254.16346	-4.4655	2.41	MIYZ	-26958.29204	-26958.29448	0.05621	2.19
M1GB	-31653.95439	-31653.78059	-4.0079	2.65	MJA0	-36343.7935	-36343.80839	0.34348	2.25
M1IF	-36662.58613	-36662.59519	0.20893	2.08	MJDB	-32135.313	-32135.22153	-2.1093	2.93
M1LC	-28940.73672	-28940.77505	0.884	2.18	MJGN	-26157.63588	-26157.60929	-0.6134	2.44
M1RC	-42626.42912	-42626.90302	10.9283	2.31	MJHU	-36759.29635	-36759.31204	0.36186	2.01
M21G	-38019.16584	-38019.12035	-1.049	2.23	MJIB	-38774.00779	-38774.00021	-0.1748	2
M2KW	-52061.52075	-52061.45376	-1.5448	2.31	MJIP	-30903.84466	-30903.95667	2.58292	2.35
M2M3	-30135.33614	-30135.31793	-0.42	1.95	MJNF	-26452.38004	-26452.30657	-1.6941	2.85
M2PU	-33088.54726	-33088.47764	-1.6055	2.13	MJRE	-39439.41648	-39438.95656	-10.606	2.17
M2WB	-30592.4351	-30592.6676	5.36162	2.4	MJUL	-34698.10642	-34698.00314	-2.3817	2.19
M31O	-47770.27585	-47770.09948	-4.0672	3.27	MJW4	-34281.03501	-34281.06742	0.74752	2.6
M31T	-35243.70269	-35243.58002	-2.8288	2.36	MJX4	-34619.29523	-34619.037	-5.9548	2.85
M33M	-27859.92381	-27859.92427	0.01054	2.14	MJXV	-31652.31368	-31652.24338	-1.6211	2.16
M342	-33790.71739	-33790.52835	-4.3593	3.27	MK3O	-31377.41235	-31377.38655	-0.5951	2.05
M36Z	-22823.97163	-22823.97351	0.04344	2.11	MK69	-30982.36633	-30982.3027	-1.4672	2.04
M38W	-33113.85696	-33113.74081	-2.6785	2.95	MKB4	-40846.35105	-40846.36949	0.42514	2.06
M3BK	-60085.89498	-60085.59087	-7.0127	2.67	MKE2	-31190.46941	-31190.36872	-2.3219	2.69
M3N5	-27545.71588	-27545.64852	-1.5534	2.25	MKE5	-31190.44251	-31190.34028	-2.3575	2.48
M3NV	-44511.76699	-44511.52885	-5.4916	2.1	MKEB	-32260.14495	-32260.0113	-3.082	2.47
M3RF	-31471.04314	-31470.9508	-2.1295	2.17	MKEJ	-44457.26297	-44456.80285	-10.611	2.75
M3RJ	-35050.86227	-35050.81037	-1.1969	2.59	MKEQ	-31190.09198	-31189.95911	-3.0641	2.52
M3RQ	-48762.38217	-48762.40973	0.63565	2.32	MKEV	-45526.81972	-45526.33228	-11.241	2.5
M3RR	-49832.19313	-49832.22225	0.6715	2.27	MKEW	-42626.74534	-42626.6291	-2.6805	2.54
M40J	-66688.75623	-66688.46798	-6.6471	2.6	MKEY	-44457.42145	-44457.35526	-1.5266	2.84

**M472**	-30506.31899	-30506.3195	0.01156	2.38	**MKEZ**	-32192.31005	-32192.18644	-2.8507	2.74
**M4CD**	-39106.34565	-39106.91043	13.024	1.91	**MKF1**	-35061.5494	-35061.25279	-6.84	2.3
**M4EP**	-25968.38726	-25968.20198	-4.2726	2.41	**MKF4**	-34897.18822	-34896.88659	-6.9557	2.3
**M4H1**	-22879.35831	-22879.3597	0.03201	1.99	**MKFO**	-41844.69733	-41844.71934	0.50754	2.58
**M4HN**	-32682.67499	-32682.935	5.99593	2.25	**MKHM**	-35205.89965	-35205.95707	1.32415	2
**M4HW**	-30838.89469	-30838.79923	-2.2013	2.66	**MKHU**	-36287.65584	-36287.847	4.40834	2.19
**M4XO**	-33948.40913	-33948.35305	-1.2932	3.69	**MKID**	-55648.55264	-55648.58104	0.65496	2.12
**M4ZJ**	-36349.93256	-36349.63331	-6.9008	2.31	**MKQE**	-34993.17821	-34993.12522	-1.2221	2.15
**M52R**	-36685.35867	-36685.15691	-4.6528	2.77	**MKR8**	-26788.6782	-26788.60503	-1.6872	2.1
**M52V**	-33310.10332	-33310.08191	-0.4936	3.13	**MKRF**	-36050.94639	-36050.73824	-4.7999	1.95
**M53K**	-31678.37567	-31678.36231	-0.3082	2.28	**MKRP**	-30649.91811	-30649.96007	0.96751	2.17
**M53R**	-31170.91093	-31170.93082	0.45883	3.1	**MKRW**	-33919.72263	-33919.72277	0.0032	1.98
**M53S**	-32240.54676	-32240.56706	0.46802	3.19	**MKU8**	-34881.62432	-34881.62851	0.0965	2.29
**M53V**	-33310.09237	-33310.08099	-0.2624	3.11	**MKW2**	-36600.7364	-36600.93916	4.67568	2.71
**M558**	-45646.98088	-45646.87582	-2.4227	2.23	**MKWN**	-40315.14291	-40315.33977	4.53973	2.28
**M565**	-33625.16516	-33624.98245	-4.2133	2.43	**ML9G**	-36538.64985	-36538.77939	2.98742	2.19
**M58C**	-33655.43249	-33655.284	-3.4243	2.59	**MLG2**	-30938.57892	-30938.7601	4.17818	2.6
**M58D**	-28073.91378	-28073.95117	0.86226	2.03	**MLII**	-30511.34303	-30511.40551	1.44091	2.44
**M58G**	-24450.4612	-24450.48078	0.45144	2.03	**MM06**	-45024.28183	-45024.4369	3.57602	2.57
**M591**	-32481.24151	-32481.22591	-0.3596	2.3	**MM3Z**	-22284.25072	-22284.07983	-3.9409	2.76
**M59L**	-31003.12921	-31002.91178	-5.0139	2.68	**MM9T**	-34731.62079	-34731.61203	-0.202	2.94
**M59Y**	-26529.41624	-26529.50255	1.99042	2.08	**MMBW**	-34998.14944	-34998.11256	-0.8506	2.35
**M5BL**	-32214.69351	-32214.78408	2.08876	2.05	**MMVK**	-31342.54507	-31342.58394	0.89624	2.22
**M5CG**	-38719.4719	-38719.31824	-3.5433	2.01	**MMVW**	-26212.24011	-26212.27879	0.8919	2.34
**M5L7**	-28601.51263	-28601.6391	2.91666	2.28	**MMWR**	-39040.0951	-39040.09922	0.09503	2.18
**M5LY**	-55626.83672	-55626.65038	-4.2971	2.41	**MN5Y**	-32090.74949	-32090.72509	-0.5627	2.88
**M5LZ**	-55626.83385	-55626.67261	-3.7182	2.29	**MN8M**	-28880.93702	-28880.87918	-1.3338	2.37
**M5O7**	-33020.93881	-33020.83269	-2.447	2.72	**MNI7**	-33731.78592	-33731.92194	3.13666	2.2
**M5OQ**	-32147.71916	-32147.7128	-0.1467	2.48	**MNN8**	-37414.90216	-37414.81049	-2.114	2.03
**M5PZ**	-55747.04992	-55747.29234	5.59027	3	**MNPF**	-28496.6916	-28497.27859	13.5362	2.14
**M5QQ**	-37997.75156	-37997.74465	-0.1592	2.86	**MNQQ**	-44087.94278	-44088.04039	2.25095	2.09
**M5UA**	-29296.99815	-29297.16691	3.89159	2.13	**MO5M**	-34881.29265	-34881.11211	-4.1634	2.92
**M62G**	-42078.58407	-42078.43944	-3.3354	2.5	**MO5P**	-32181.46303	-32181.21849	-5.6394	2.95
**M639**	-34549.22231	-34549.13109	-2.1036	3.91	**MO8M**	-33470.66419	-33470.30396	-8.307	2.57
**M64A**	-31045.18141	-31044.96913	-4.8953	2.39	**MO9U**	-37026.89955	-37027.00248	2.37357	2.37
**M658**	-35749.15163	-35749.28222	3.01143	2.38	**MOA5**	-23157.61857	-23157.64767	0.67109	2.15
**M69E**	-34150.70154	-34150.75662	1.27014	2.09	**MOAY**	-37343.59852	-37343.87991	6.48903	2.32
**M6A7**	-39465.86907	-39465.83699	-0.7398	2.24	**MOGO**	-32192.58353	-32192.57852	-0.1156	2.19
**M6DM**	-35309.76091	-35309.66238	-2.272	2.48	**MOSI**	-40606.53284	-40606.7795	5.68821	2.3
**M6IT**	-49435.62916	-49435.58126	-1.1045	1.97	**MOVX**	-42079.89471	-42079.92924	0.79631	2.14
**M6XP**	-42085.31432	-42085.14797	-3.836	2.76	**MOZ8**	-29704.59162	-29704.60587	0.32844	3.33
**M6YE**	-34397.16747	-34396.8769	-6.7006	2.63	**MP07**	-29964.56674	-29964.58283	0.37096	2.86
**M704**	-43071.72312	-43071.75983	0.84653	2.84	**MP0W**	-28406.27506	-28406.41073	3.12859	2.36
**M73Z**	-35965.07112	-35965.03544	-0.8229	2.3	**MP0X**	-44126.94449	-44126.97745	0.7599	2.51
**M74E**	-35997.76729	-35997.732	-0.8137	2.24	**MPDZ**	-40129.28022	-40129.2188	-1.4164	2.03
**M74F**	-36997.83531	-36997.84869	0.30848	2.72	**MPFN**	-21918.90094	-21918.95027	1.13762	2.05

**M756**	-31713.52676	-31713.44132	-1.9701	2.72	**MPM1**	-35976.92406	-35976.88162	-0.9787	2.99
**M75Q**	-57285.40329	-57285.39139	-0.2746	2.07	**MPQ6**	-42112.44897	-42112.40206	-1.0819	2.42
**M75T**	-30331.53042	-30331.56835	0.87473	2.29	**MPQI**	-46825.93573	-46826.23106	6.81063	2.41
**M76N**	-38086.01886	-38086.60946	13.6195	2.1	**MPU6**	-29475.89201	-29475.87962	-0.2858	2.25
**M778**	-45150.44179	-45150.39172	-1.1545	2.4	**MPUU**	-43149.86847	-43149.71401	-3.562	2.73
**M77V**	-28868.29547	-28868.31617	0.47736	2.23	**MPUX**	-32176.01224	-32176.0001	-0.28	2.43
**M7BF**	-30974.81198	-30974.70481	-2.4714	2.76	**MPV5**	-37220.47015	-37220.34147	-2.9675	2.1
**M7H2**	-49036.76619	-49036.75392	-0.2829	2.9	**MPY1**	-23777.43097	-23777.56857	3.17317	2.49
**M7JV**	-25934.31514	-25934.22281	-2.1292	2.14	**MPZ4**	-23204.3463	-23204.33188	-0.3326	2.01
**M7KW**	-27389.92781	-27389.84066	-2.0097	2.83	**MPZK**	-27383.0168	-27383.00321	-0.3134	2.25
**M7LL**	-28007.0872	-28007.19249	2.42801	2.35	**MQ0I**	-44759.34482	-44759.44225	2.24682	2.28
**M7OG**	-32645.87802	-32645.7833	-2.1844	2.24	**MQ22**	-34553.84942	-34553.39157	-10.558	2.38
**M7OP**	-36565.79028	-36565.85024	1.38284	2.86	**MQ27**	-30103.45906	-30103.4594	0.0078	2.08
**M7OY**	-49071.59215	-49071.61466	0.51925	2.92	**MQ4M**	-43149.08703	-43148.97219	-2.6482	2.21
**M7U2**	-33969.56248	-33969.50726	-1.2735	2.48	**MQAQ**	-32852.40873	-32852.42639	0.40716	2.23
**M7W7**	-23385.80456	-23385.76541	-0.9027	2	**MQC2**	-26337.74212	-26337.84752	2.43056	2.4
**M82G**	-32523.44616	-32523.40579	-0.931	2.56	**MQCR**	-37822.13558	-37821.71589	-9.6783	2.65
**M84N**	-21814.63025	-21814.61548	-0.3407	2.03	**MQD4**	-43586.66507	-43586.40503	-5.9965	3.11
**M86E**	-54577.98128	-54577.67551	-7.0513	2.45	**MQF9**	-37893.7518	-37893.79169	0.91986	2.2
**M890**	-27003.52053	-27003.3979	-2.828	2.55	**MQFX**	-38328.42834	-38328.42868	0.00804	2.29
**M89C**	-27543.95899	-27543.85084	-2.494	2.52	**MQLZ**	-29070.286	-29070.26576	-0.4666	1.84
**M8F0**	-29764.20966	-29764.16608	-1.0051	2.1	**MQML**	-43689.68991	-43689.70005	0.23383	2.17
**M8F7**	-28088.73299	-28088.72097	-0.2772	2.15	**MQOQ**	-45863.35298	-45863.30596	-1.0843	2.83
**M8F9**	-29763.91609	-29763.95221	0.83286	2.19	**MQQ3**	-30122.14939	-30122.14801	-0.0318	2.8
**M8FX**	-46089.01106	-46088.97574	-0.8146	2.13	**MQQ9**	-25368.36963	-25368.33801	-0.7291	2.2
**M8GR**	-34827.33405	-34827.35525	0.48876	2.09	**MQRO**	-30239.31136	-30239.32948	0.41781	2.07
**M8GX**	-35263.62594	-35263.68557	1.37503	2.02	**MQTC**	-37523.62254	-37523.70883	1.98993	2.85
**M8IP**	-23843.35197	-23843.38549	0.77287	2.31	**MQUQ**	-43149.89121	-43149.83133	-1.3808	2.28
**M8J7**	-25579.25956	-25579.26416	0.10615	2.19	**MQVC**	-25223.70043	-25223.69731	-0.0721	2.27
**M8LN**	-45515.92005	-45515.69947	-5.0867	3.62	**MQW1**	-43586.54921	-43586.47849	-1.6308	2.48
**M8NR**	-32789.74315	-32789.7563	0.30338	2.15	**MQXY**	-28127.53278	-28127.53044	-0.054	2.09
**M8P3**	-24429.76206	-24429.81665	1.25895	2.17	**MR41**	-41545.03625	-41544.92492	-2.5674	2.11
**M8PT**	-35755.71287	-35756.05103	7.79794	2.34	**MR7F**	-44251.72541	-44251.7894	1.47563	2.74
**M8R4**	-33890.44605	-33890.48783	0.96349	2.3	**MR8F**	-28279.73678	-28279.83847	2.34504	2.02
**M8X2**	-35690.36048	-35690.15272	-4.7911	2.87	**MRG4**	-26855.19652	-26855.12565	-1.6343	2.07
**M8X5**	-35794.83883	-35794.64254	-4.5264	2.46	**MRH8**	-36745.23855	-36745.21708	-0.495	2.63
**M8XR**	-33918.37946	-33918.34646	-0.761	3.03	**MRI3**	-34406.19367	-34406.25447	1.40196	2.16
**M8XX**	-31778.25783	-31777.82775	-9.9177	2.73	**MRIZ**	-30670.68981	-30671.22541	12.351	2.32
**M8ZP**	-34769.78562	-34769.81488	0.67487	1.97	**MRJ9**	-30670.54714	-30671.2254	15.641	2.37
**M93F**	-31902.8095	-31902.86797	1.34816	2.05	**MRKZ**	-33115.18472	-33115.22467	0.92114	2.29
**M985**	-30107.86465	-30107.85758	-0.1631	2.33	**MRLR**	-46761.61224	-46761.86844	5.90789	2.75
**M9IP**	-26958.91549	-26958.94765	0.74145	2.31	**MROD**	-34009.55821	-34009.55515	-0.0705	2.18
**M9J2**	-47770.32864	-47770.15084	-4.1003	3.05	**MRQB**	-26497.61051	-26497.56689	-1.006	2.16
**M9L0**	-37253.59253	-37253.41374	-4.1231	2.26	**MRTM**	-26623.04133	-26622.90515	-3.1403	2.71
**M9LX**	-33342.03193	-33342.02908	-0.0657	2.15	**MS4O**	-28390.07351	-28390.0509	-0.5213	2.18
**M9S5**	-33120.61644	-33120.60584	-0.2445	2.67	**MS9H**	-32149.61918	-32149.48992	-2.9807	2.1

M9TO	-30941.77582	-30941.71623	-1.3743	2.82	MSDL	-33482.08054	-33481.94908	-3.0316	2.06
M9WI	-33511.58689	-33511.60096	0.32453	4.22	MSGH	-28964.62464	-28964.63142	0.15632	2.13
M9XT	-29304.83385	-29304.7776	-1.2973	2.34	MSOZ	-42429.5271	-42429.40795	-2.7477	3.34
MA6Y	-29666.15632	-29666.19131	0.80701	2.46	MSUU	-26294.17515	-26294.10371	-1.6474	2.48
MA82	-37345.02668	-37345.13001	2.38287	2.33	MSWF	-28144.8678	-28144.83425	-0.7738	2.29
MABZ	-42875.91583	-42875.9225	0.15375	2.08	MSZV	-37153.56595	-37153.90126	7.73232	2.16
MAIE	-21345.77452	-21345.85725	1.90781	2.48	MT8Z	-31503.33478	-31503.18385	-3.4807	0.91
MAJL	-31248.85399	-31248.78031	-1.6992	2.36	MT9I	-35674.31367	-35674.16937	-3.3275	1.82
MAQI	-30089.11878	-30089.28299	3.7866	2.37	MTGM	-36717.39561	-36717.74905	8.15033	2.22
MAQV	-32287.50965	-32287.25436	-5.8872	2.12	MTL8	-24923.97819	-24923.80722	-3.9427	2.02
MB1I	-32300.37611	-32300.37545	-0.0152	2.32	MTLQ	-25987.94015	-25987.94028	0.00285	2.48
MB2D	-34520.36056	-34520.21894	-3.2657	2.2	MTPB	-28492.89863	-28492.91078	0.28025	3.42
MB6A	-29099.17233	-29099.13994	-0.7471	2.1	MTU7	-48496.04757	-48496.06969	0.51021	2.14
MB7V	-44026.96882	-44026.83599	-3.0631	2.39	MTUQ	-45188.80453	-45188.74323	-1.4136	2.19
MBA8	-44184.41892	-44184.86868	10.3718	2.84	MTVT	-33351.25445	-33351.2934	0.89812	2.18
MBGE	-29534.37761	-29534.55769	4.15272	2.16	MU03	-28177.27265	-28177.36982	2.24058	2.07
MBI3	-34223.03559	-34222.97289	-1.446	2.65	MU06	-36301.26202	-36301.20901	-1.2224	2.57
MBJM	-27664.13561	-27663.98361	-3.5052	2.17	MU1U	-42608.29982	-42608.32396	0.55674	1.99
MBVI	-30603.76152	-30603.75384	-0.1771	2.11	MU26	-31204.88866	-31204.89802	0.21578	1.91
MBX2	-35221.22219	-35221.22456	0.05466	2.37	MU75	-36301.22058	-36301.24004	0.44877	2.01
MBYB	-38454.97422	-38455.15183	4.09577	2.61	MU86	-31113.82671	-31113.83337	0.15346	2.02
MC1W	-55616.07387	-55616.02007	-1.2407	2.08	MU8I	-31113.83784	-31113.85107	0.30519	2.31
MC1Y	-24351.32795	-24351.33976	0.27252	2.64	MU8Y	-31113.9092	-31113.8411	-1.5704	2.08
MC3Z	-32141.68857	-32141.71402	0.58703	3.55	MUDV	-45612.47155	-45612.47598	0.1021	2.22
MC8H	-37309.90982	-37309.57921	-7.6241	2.67	MUES	-34243.52297	-34243.4504	-1.6733	2.12
MCD9	-42064.65906	-42064.65824	-0.019	2.39	MUGI	-38137.62179	-38137.61972	-0.0477	2.27
MCF9	-32573.39211	-32573.74302	8.09205	2.46	MUNQ	-32122.47037	-32122.46795	-0.0559	2.32
MCIW	-42064.57572	-42064.57989	0.09635	2.55	MUTV	-29953.89336	-29953.82738	-1.5215	2.33
MCL3	-30505.05303	-30505.07149	0.42567	2.33	MUU0	-37683.09666	-37683.72079	14.3927	2.13
MCQF	-44609.83651	-44609.87913	0.98288	2.55	MUVF	-36232.90901	-36232.82095	-2.0306	2.01
MCRH	-36489.06015	-36489.08852	0.65413	2.32	MUWB	-35253.4289	-35253.71461	6.58871	2.32
MCT5	-32225.43308	-32225.54597	2.60331	2.62	MUWM	-34414.64698	-34414.59715	-1.149	2.72
MCX9	-36986.8048	-36986.64045	-3.7901	3.23	MUYM	-33187.05327	-33186.86352	-4.3755	2.76
MCXZ	-45199.48566	-45199.32334	-3.7432	3.15	MVHS	-59384.708	-59384.59011	-2.7186	3.2
MD05	-70643.69685	-70643.59137	-2.4324	2.49	MVJQ	-29047.19513	-29047.06006	-3.1147	2.05
MD1F	-35319.62888	-35319.43605	-4.4468	2.36	MVM3	-36480.05613	-36480.05629	0.00354	2.37
MD97	-33795.77689	-33796.20744	9.92875	2.35	MVN8	-57859.77182	-57859.21345	-12.876	2.86
MD9D	-32235.45172	-32235.58747	3.13046	2.98	MVOR	-34457.79621	-34458.13558	7.82595	2.14
MD9M	-29616.14749	-29616.42222	6.3354	2.12	MVOZ	-28629.27091	-28629.11051	-3.6989	2.11
MD9V	-28143.14936	-28143.34763	4.5722	2.35	MVPS	-32897.78887	-32897.75262	-0.836	3.63
MDAF	-31614.04545	-31613.60907	-10.063	2.29	MVU3	-25142.76182	-25142.57364	-4.3394	2.12
MDVF	-30631.50813	-30631.58739	1.82778	2.41	MVX2	-34551.50292	-34551.39726	-2.4366	2.23
MDXB	-36487.79384	-36488.97335	27.2003	2.11	MW08	-35576.74367	-35577.09211	8.03529	2.05
MDXV	-33350.35613	-33350.38268	0.61208	2.31	MW6Y	-34149.82262	-34149.85903	0.83959	2.24
ME3B	-33162.26858	-33162.30222	0.77562	2.58	MW7C	-32135.31265	-32135.29586	-0.3871	2.7
ME75	-31144.32966	-31144.47004	3.23725	2.38	MW7D	-32135.29129	-32135.29071	-0.0133	2.57

**ME82**	-30299.74009	-30299.73567	-0.1019	3.25	**MW7G**	-27955.60541	-27955.5033	-2.3548	2.5
**MECV**	-38446.48419	-38446.26616	-5.028	2.33	**MW7H**	-29019.67727	-29019.67134	-0.1367	2.59
**MEDF**	-33748.33167	-33748.17171	-3.6888	2.92	**MW7I**	-32135.38626	-32135.28464	-2.3434	2.92
**MEHW**	-29625.81971	-29625.81978	0.00173	2.09	**MW7L**	-32135.2774	-32135.23783	-0.9124	2.34
**MEJE**	-31825.324	-31825.32412	0.00263	2.09	**MW8U**	-47092.95899	-47092.78916	-3.9164	3.18
**MEK2**	-54017.25275	-54017.25076	-0.0458	2.01	**MWAP**	-32309.53548	-32309.51726	-0.4201	2.44
**MEKB**	-31116.0266	-31116.07869	1.20118	2.12	**MWAU**	-32746.10953	-32746.06363	-1.0585	2.36
**MEO8**	-31510.27204	-31510.2054	-1.5366	2.13	**MWDD**	-28882.24803	-28882.07703	-3.9434	1.28
**MER9**	-29152.69216	-29153.30386	14.1059	2.74	**MWJK**	-35186.3477	-35186.43704	2.06041	2.61
**MEW2**	-42001.38998	-42001.35716	-0.7568	2.35	**MWNH**	-37099.30197	-37099.51523	4.91776	2
**MEWN**	-35996.03916	-35995.97603	-1.4558	1.95	**MWRO**	-34009.69208	-34009.58217	-2.5346	2.78
**MEXJ**	-39268.36399	-39268.317	-1.0836	2.65	**MWUZ**	-54123.53378	-54123.44493	-2.0489	2.09
**MEZQ**	-33232.71478	-33232.70302	-0.2712	2.02	**MWXJ**	-25693.12355	-25692.96565	-3.6411	2.06
**MF29**	-32472.7208	-32472.57372	-3.3919	2.14	**MWY0**	-33699.49094	-33699.50371	0.29442	2.14
**MF44**	-16684.9345	-16684.95756	0.53175	2.2	**MWY5**	-36576.7134	-36576.88155	3.87756	2.4
**MF6J**	-32313.72311	-32313.8029	1.8401	2.55	**MX3K**	-23314.59201	-23314.57019	-0.503	2.06
**MF7D**	-36911.57705	-36911.62523	1.11083	2.03	**MX3L**	-35723.45663	-35723.3488	-2.4866	2.34
**MF80**	-29600.016	-29600.11681	2.3246	2.65	**MX45**	-34255.22788	-34255.2229	-0.1147	2.21
**MFBY**	-35978.00581	-35977.94129	-1.4879	2.03	**MX7G**	-35055.05965	-35055.04462	-0.3466	2.37
**MFEZ**	-31482.77786	-31483.26102	11.142	3.1	**MX8G**	-47560.94689	-47560.82563	-2.7964	2.78
**MFFS**	-40469.31948	-40469.38292	1.46303	2.19	**MXH9**	-40534.15037	-40534.32169	3.95062	2.41
**MG2Y**	-66688.57811	-66688.53595	-0.9722	2.58	**MXHW**	-50530.66659	-50530.71982	1.22749	2.39
**MG3X**	-34554.78165	-34554.72004	-1.4207	2.21	**MXJX**	-35996.03118	-35996.61051	13.3598	2.84
**MG5C**	-34528.59804	-34528.54768	-1.1614	2.44	**MXMZ**	-30213.18016	-30213.18349	0.07676	2.81
**MG8J**	-32676.83677	-32676.86687	0.69397	1.99	**MXRO**	-28419.06313	-28418.84928	-4.9317	2.32
**MG9K**	-34668.87866	-34668.84719	-0.7258	2.28	**MXT3**	-33216.59735	-33217.05974	10.6628	2.44
**MGB3**	-23991.33238	-23991.05827	-6.3211	2.02	**MXXZ**	-33304.84685	-33304.77152	-1.737	2.13
**MGB7**	-26005.59883	-26005.45766	-3.2554	2.38	**MY4T**	-23046.1722	-23046.17107	-0.0261	2.56
**MGD5**	-45515.91483	-45515.59797	-7.307	3.22	**MY5J**	-40349.76945	-40349.74087	-0.659	2.14
**MGPW**	-37069.99159	-37069.77116	-5.0833	2.28	**MY81**	-29541.43438	-29541.32871	-2.4369	2.18
**MGYO**	-27499.54064	-27499.71323	3.97983	2.04	**MYE5**	-27935.52039	-27935.52224	0.04278	2.36
**MGYW**	-37611.21578	-37611.25718	0.95455	2.96	**MYJ4**	-31985.866	-31985.85002	-0.3685	2.07
**MH3E**	-31541.49526	-31541.41173	-1.9264	2.49	**MYNS**	-35181.77721	-35181.62909	-3.4158	3.99
**MH3H**	-29605.53846	-29605.48068	-1.3324	2.42	**MYTD**	-29268.76693	-29268.67959	-2.014	2.29
**MH3N**	-32682.50595	-32682.37549	-3.0086	2.96	**MYTU**	-37569.38844	-37569.43318	1.03177	2.08
**MH3P**	-92800.75988	-92800.77214	0.28275	2.5	**MZ18**	-34066.07189	-34066.3251	5.83921	2.85
**MH3Q**	-28595.23551	-28595.20073	-0.8021	2.36	**MZKW**	-48780.89405	-48780.73273	-3.7203	2.27
**MH5F**	-28259.81383	-28259.19302	-14.316	2.25	**MZM4**	-34784.14342	-34784.18681	1.00068	2.02
**MH5I**	-42635.04926	-42634.77935	-6.2241	2.58	**MZP2**	-31158.40833	-31158.39425	-0.3248	2.41
**MH7P**	-45538.52458	-45538.53329	0.20074	2.34	**MZUI**	-47804.32469	-47804.24797	-1.7692	2.94
**MHCJ**	-38246.55446	-38246.41845	-3.1364	3.04					

Table S4. Overall performance of Confgen and Auto3D in reproduction of model/ideal structures from ideal structures using several cut-off values set for success percentage (%).

				wrt/ QM optimized model							wrt/ QM optimized ideal						
Method	Parset	RMSD	size	success %				mean	min	max	success %				mean	min	max
				≤0.5Å	≤1.0 Å	≤1.5 Å	≤2.0 Å				≤0.5Å	≤1.0 Å	≤1.5 Å	≤2.0 Å			
Auto3D	sdf input	all	62	43.5	77.2	92.3	98.9	0.69	0.06	2.45	65.4	92.9	99.3	100.0	0.47	0.03	1.80
		top 1		7.5	18.2	31.2	53.5				7.5	17.3	33.7	60.1			
		top 3		15.0	35.1	55.4	79.3	1.36	0.06	4.11	18.2	38.3	63.1	85.9	1.24	0.03	3.39
		top 10		28.2	57.6	77.4	92.9	0.98	0.06	3.66	40.5	68.8	89.7	96.4	0.78	0.03	2.66
DeepConf	parset6	all	21	23.5	59.9	82.9	94.3	0.93	0.10	3.10	36.7	84.7	94.1	97.0	0.66	0.11	2.35
		top 1		4.8	14.4	23.9	45.3				8.4	22.6	41.7	68.6			
		top 3		9.3	27.3	48.5	74.0	1.51	0.12	3.93	19.6	49.7	77.9	92.9	1.06	0.12	3.08
		top 10		21.0	50.1	73.3	92.3	1.09	0.12	3.10	33.3	76.3	91.8	97.7	0.76	0.08	2.54
	parset7	all	207	37.1	77.0	93.8	98.4	0.71	0.07	2.27	58.8	95.7	99.3	99.8	0.49	0.06	2.00
		top 1		4.8	14.4	23.9	45.3				8.4	22.6	41.7	68.6			
		top 3		9.1	25.3	44.4	69.7	1.57	0.08	3.85	17.1	45.1	72.0	89.5	1.15	0.10	3.10
		top 10		18.0	45.3	67.0	88.2	1.19	0.08	3.56	33.0	70.4	88.8	97.0	0.82	0.06	2.52
	parset2	all	18	35.5	68.3	87.2	97.3	0.81	0.06	2.53	54.7	88.6	96.4	99.3	0.54	0.03	2.24
		top 1		9.8	20.7	36.2	57.2				9.6	17.5	29.4	56.5			
		top 3		21.6	37.8	62.6	82.9	1.25	0.06	3.59	20.3	40.1	62.6	87.2	1.21	0.03	3.13
		top 10		31.7	58.8	81.1	94.8	0.92	0.06	2.89	43.1	76.8	90.7	98.4	0.70	0.03	2.47
	parset5	all	36	46.6	74.9	89.8	97.2	0.69	0.02	2.32	67.5	92.2	98.9	99.6	0.43	0.03	1.59
		top 1		13.4	24.0	37.8	60.8				7.4	16.6	30.0	50.5			
		top 3		23.0	40.6	62.5	82.3	1.23	0.05	3.96	18.7	35.3	57.2	82.0	1.28	0.04	3.18
		top 10		34.6	58.3	77.7	91.9	0.93	0.04	3.00	40.6	71.0	89.0	97.2	0.72	0.04	2.34
	parset4	all	322	56.5	88.3	97.0	99.5	0.54	0.03	2.38	71.0	96.7	99.5	99.8	0.39	0.02	2.01
		top 1		10.3	22.9	38.3	57.9				5.8	15.0	26.6	53.3			
		top 3		20.6	41.1	59.1	76.6	1.31	0.03	3.92	12.9	29.7	47.4	72.9	1.46	0.06	3.37
		top 10		32.7	57.5	75.9	89.0	1.01	0.03	3.92	25.9	50.2	74.8	90.7	1.04	0.04	3.09
RDKit	MMFF94	all	20	35.8	72.2	87.9	97.7	0.78	0.05	2.88	47.8	87.5	95.9	99.8	0.59	0	2.05
		top 1		6.8	18.2	32.1	60.8				5.9	15.9	27.3	54.0			
		top 3		15.5	35.3	56.7	83.1	1.33	0.05	3.36	16.4	38.5	58.5	83.8	1.28	0.05	3.38
		top 10		29.6	59.7	78.4	94.5	0.95	0.05	3.14	37.8	72.7	89.1	98.4	0.76	0.05	2.58
	UFF	all	20	29.4	72.2	89.1	97.7	0.82	0.06	2.78	38.7	85.0	96.6	99.5	0.65	0.05	2.42
		top 1		8.0	22.8	38.7	60.6				5.2	13.7	23.7	46.7			
		top 3		14.8	40.8	62.0	84.1	1.27	0.06	3.58	13.9	34.9	53.3	76.3	1.39	0.05	3.41
		top 10		24.6	62.0	82.7	96.1	0.93	0.06	3.07	30.3	71.8	90.9	97.9	0.79	0.05	2.50

Table S5. Effect of maximum step / convergence threshold on conformer generation performances.

	auto3d_f002			auto3d_f00001			confgen_f002			confgen_f00002		
	Auto3D global to G16 global			Auto3D global to G16 global								
ID	#auto3d_numconf	min top	min in all	#auto3d_numconf	min top	min in all	parset5	mıntop	min in all	parset5	mıntop	min in all
38W	26	1.1	0.6	13	1.1	0.8	53	1.1	0.9	23	1.1	1.1
2WB	28	0.8	0.6	15	0.8	0.8						
2M3	117	2.2	0.9	68	2.8	0.7	48	2.2	1.3	34	2.5	1.1
2KW	35	2.6	1.0	6	2.6	1.1	48	2.2	1.0			
21G	22	0.5	0.5	20	0.4	0.4	34	0.5	0.3			
1GB	105	2.5	1.0	28	1.1	1.1	47	2.1	1.6	27	2.1	1.6
0YO	4	1.5	0.1	2	1.5	1.5	7	1.5	0.1	10	1.4	0.1
0VG	69	2.7	1.1	25	2.7	1.9	43	2.8	1.6			
0C7	99	0.6	0.6	31	0.5	0.2				36	0.5	0.2
05B	13	1.0	0.7	9	0.9	0.7	11	1.8	1.6	10	1.6	1.5
mean	51.8	1.6	0.7	21.7	1.4	0.9	36.4	1.8	1.0	23.3	1.5	0.9
%success<0.5		1	2		2	2		1	2		1	2
%success<1		3	9		4	6		1	3		1	2
%success<1.5		6	10		7	9		3	5		3	4

Table S6. Computational performance of Auto3D and DeepConf with different parameter settings for **one ligand** with **five** torsion points.

	parset1	parset2	parset3	parset4	parset5	parset6	parset7	auto3d
Number of SPE calculations	10240	320	320	10240	320	320	320	32
Number of optimizations	1024	32	64	1024	32	32	0	32
Number of clusters	1024	32	32	1024	64	32	32	0
Wall time (minutes)*	40	2	3.5	80	8	4	0.2	1.2
*: The calculations are performed on the CPU only using a single machine with Intel(R) Xeon(R) Platinum 8480+ 2.0GHz, 256GB Memory, 2x56=112 cores								
